# Effects and moderator of high-intensity interval training and moderate-intensity continuous training among children and adolescents with overweight or obese: a systematic review and meta-analysis

**DOI:** 10.3389/fphys.2025.1625516

**Published:** 2025-07-30

**Authors:** Weihua Zheng, Mingyue Yin, Yan Guo, Haiyang Liu, Jiaxin Sun, Ao Zhu, Yuming Zhong, Kai Xu, Hansen Li, Shunzhe Piao

**Affiliations:** ^1^School of Social Sports, Shenyang Sport University, Shengyang, China; ^2^School of Athletic Performance, Shanghai University of Sport, Shanghai, China; ^3^Sport Institute, Hua Qiao University, Quanzhou, China; ^4^School of Physical Education, Sichuan Agricultural University, Ya’an, China

**Keywords:** HIIT (high intensity interval training), MICT, children, adolescent, body shape, cardiopulmonary health

## Abstract

**Objectives:**

This meta-analysis aimed to synthesize current evidence and address inconsistencies in the effects of high-intensity interval training (HIIT) and moderate-intensity continuous training (MICT) on children and adolescents with overweight or obesity. We sought to: (1) assess the effects of HIIT and MICT *versus* non-exercise controls; (2) compare HIIT and MICT directly; and (3) identify potential moderators through subgroup analyses.

**Methods:**

Systematic searches were conducted in PubMed, Web of Science, China National Knowledge Infrastructure, and CSTJ. Standardized mean differences (SMD) were calculated using a random-effects model. Subgroup and sensitivity analyses explored potential moderators.

**Results:**

A total of 26 moderate-to-high-quality studies (Randomized controlled trials and non-RCT) involving 1,078 participants (765 males, 313 females; aged 9–19) were included. Compared with controls, HIIT significantly reduced fat mass (SMD = −0.69), waistline (SMD = −0.67), body weight (SMD = −0.81), diastolic blood pressure (diastolic blood pressure, SMD = −0.68), and improved VO_2_max (SMD = 2.06). moderate-intensity continuous training showed significant effects on BMI (SMD = −1.58), body weight (SMD = −0.59), DBP (SMD = −0.60), and VO_2_max (SMD = 1.26). HIIT outperformed MICT in improving VO_2_max (SMD = 0.81) and reducing systolic blood pressure (systolic blood pressure, SMD = −0.51). Subgroup analyses showed that HIIT yielded greater benefits than MICT in improving VO_2_max among overweight children, reducing SBP in obese male adolescents, and in programs involving more than three sessions per week.

**Conclusion:**

HIIT was more effective than MICT in improving VO_2_max and reducing SBP, especially in specific subgroups. Running-based HIIT three times per week is recommended, while cycling may offer a safer alternative. Results should be interpreted cautiously due to limited subgroup data and potential bias.

**Prospero Registration:**

CRD42024612098.

## 1 Introduction

The World Health Organization (WHO) defines overweight and obesity as “abnormal or excessive fat accumulation that may impair health” (Murray et al., 2020). These conditions are associated with an increased risk of chronic diseases such as cardiovascular disease, diabetes, hypertension, and various forms of cancer (Koyuncuoğlu Güngör, 2014). As of 2022, WHO reports that more than 390 million children and adolescents aged 5–19 years worldwide are affected by overweight or obesity (Okunogbe et al., 2022). The high prevalence of overweight or obesity among children and adolescents has become a major global public health concern. Evidence indicates that children and adolescents with overweight or obesity are more likely to remain obese in adulthood compared to their peers with healthy weight (Simmonds et al., 2015; 2016; Llewellyn et al., 2016), The elevated risk of obesity is associated with a range of contributing factors, including insufficient physical activity, unhealthy dietary habits, smoking, excessive alcohol consumption, and sedentary behavior (Cureau et al., 2018). Among these, insufficient physical activity has been identified by the WHO and numerous health research institutions as one of the most significant global risk factors affecting health. It can lead to serious adverse effects on both physical and mental wellbeing.

Exercise is a necessary approach to increase physical activity levels and thereby promote both physical and mental health (Bull et al., 2020). Traditional forms of exercise typically involve longer-duration exercise, Common forms of exercise such as jogging and cycling are typically performed as moderate-intensity continuous training (MICT). Although previous studies have demonstrated that MICT can significantly improve body mass index (BMI) and cardio respiratory fitness in children and adolescents with overweight or obesity (Thorogood et al., 2011; O’Donoghue et al., 2021), modern youth often face academic pressures and an increasing array of leisure-time activities (Larson and Verma, 1999; Stiglic and Viner, 2019). These factors, along with limited access to exercise spaces and insufficient time, may pose significant barriers to regular participation in physical activity (Kimm et al., 2006). Therefore, there is a growing need for exercise-based health strategies that are both effective and time-efficient for children and adolescents with overweight or obesity.

High-intensity interval training (HIIT) has gained widespread popularity as an efficient form of exercise. It is defined as a type of intermittent training involving short bursts of high-intensity activity alternated with periods of rest or low-intensity exercise (Coates et al., 2023). HIIT can take various forms, including cycling, treadmill running, body weight exercises, and small-area games. Wewege et al. (2017) found that HIIT achieved comparable improvements in body composition in adults to MICT while requiring less than 40% of the training time. In a comprehensive systematic review, Poon et al. (2024a) also concluded that HIIT leads to a greater reduction in body fat percentage (BF%) compared to MICT in healthy adults, with a weighted mean difference (WMD) of −0.77. Yin et al. (2024a) further reported that low-volume HIIT (lv-HIIT) is an effective and time-efficient strategy for improving cardiorespiratory fitness and metabolic health in non-athlete adults. Notably, Yin et al. (2023) also found that HIIT achieved similar improvements in maximal fat oxidation capacity compared to MICT, despite requiring significantly less time commitment.

Nevertheless, while previous findings primarily highlight the benefits of HIIT over MICT in adults, a review of the literature reveals ongoing controversy regarding their comparative effects on body composition and metabolic health in children and adolescents. In terms of body composition, HIIT produced more significant improvements compared to MICT (Miguet et al., 2020; Julian et al., 2022). However, Leite et al. (2022) and Koubaa, (2013) reported that MICT was more effective than HIIT in improving body composition. Moreover, Su et al. (2024) found no significant difference between HIIT and MICT in terms of body composition improvements. Regarding glucose and lipid metabolism, Cao et al. (2022a) observed that HIIT was particularly effective in reducing triglyceride levels, while MICT demonstrated more stable improvements in total cholesterol and low-density lipoprotein (LDL) cholesterol (Cao et al., 2021). Nevertheless, some studies reported no significant differences between HIIT and MICT in their effects on glucose and lipid metabolism (Dias et al., 2018; Liu et al., 2020; Yin et al., 2020). In terms of blood pressure, earlier studies suggested that HIIT significantly reduced systolic blood pressure (SBP) in overweight and obese adolescents, but had no effect on diastolic blood pressure (DBP) (García-Hermoso et al., 2016). In contrast, Leite’s study showed that HIIT induced a significant reduction in DBP, indicating a potential advantage over MICT in this regard (Leite et al., 2022). These inconsistencies underscore the need for more conclusive evidence on this topic. In theory, systematic reviews and meta-analyses offer stronger evidence than individual controlled trials. Currently, most reviews focusing on children and adolescents suggest that HIIT is more effective than MICT in improving cardiorespiratory fitness (Cao et al., 2019; Deng and Wang, 2024). However, there remains disagreement regarding outcomes related to body composition and cardiometabolic health. For instance, Wang et al. (2024) reported that HIIT was superior to MICT in improving body composition and cardiometabolic indicators. In contrast, Yin et al. (2020) conducted a meta-analysis examining the effects of HIIT and MICT on health outcomes in children and adolescents, finding that HIIT had superior effects on VO_2_peak, there were no significant differences in body composition or cardiometabolic parameters. Furthermore, we observed that some studies did not further investigate the sources of heterogeneity (Wang et al., 2024), and others combined data from both obese and non-obese populations in the analysis, which may have compromised the reliability of the results (Yin et al., 2020). Moreover, several key questions remain unanswered. For instance, do these interventions exert different effects in healthy *versus* overweight or obese adolescents? How do the outcomes compare to those observed in non-exercise control groups? Do variables such as sex, intervention duration, frequency, or training modality moderate the intervention effects? Addressing these questions is essential for the development of personalized exercise prescriptions tailored to individual characteristics.

Therefore, this meta-analysis aims to systematically synthesize existing research evidence and address these inconsistencies and gaps by applying rigorous statistical methods and stratified analyses. Specifically, we seek to answer three key questions:1) To evaluate the intervention effects of HIIT and MICT on children and adolescents with overweight or obesity compared to a non-exercise control group. 2) To examine the differences in intervention effects between HIIT and MICT when directly compared in this population. 3) To identify potential moderators that may influence the observed outcomes through subgroup analyses.

## 2 Methods

### 2.1 Search strategy

This meta-analysis was conducted and reported following the 2020 PRISMA (Preferred Reporting Items for Systematic Reviews and Meta-Analyses) statement (Parums, 2021), and the methodological quality was rigorously self-assessed using the A Measurement Tool to Assess Systematic Reviews 2 (AMSTAR 2) checklist. In line with the principles of open science and to enhance the transparency and reproducibility of this study, the review protocol was prospectively registered in the PROSPERO database. A comprehensive literature search was conducted in PubMed, Web of Science, China National Knowledge Infrastructure (CNKI), and China Science and Technology Journal Database (CSTJ) from their inception to 10 January 2025. To ensure the completeness of included studies, we also manually searched the reference lists of included articles, their subsequent citations, and “related articles” suggested by PubMed and Web of Science. Detailed search terms are provided in Supplementary Table 1–2.

### 2.2 Inclusion and exclusion criteria

Eligibility criteria were predefined based on the PICOS framework (Population, Intervention, Comparison, Outcomes, and Study design). No time restriction was applied during the literature search to maximize the inclusion of all potentially relevant studies; subsequent strict screening based on predefined criteria ensured the relevance and quality of the included studies, thereby supporting the comprehensiveness and validity of the findings. 1) Population: According to the WHO 2007 Growth Reference for children and adolescents aged 5–19 years (De Onis, 2007), this review included children (5–12 years)and adolescents (13–19 years). Overweight was defined as a body mass index (BMI) at or above the 85th percentile, and obesity as a BMI at or above the 97th percentile, based on age- and sex-specific reference populations. Animal studies were excluded during the title/abstract screening phase. 2) Intervention: MICT was defined as exercising at 64%–76% of maximum heart rate (HRmax), or 46%–63% of maximal oxygen uptake (VO_2_max), or 40%–59% of heart rate reserve (HRR), or with a perceived exertion rating (RPE) of 12–13 (Williams et al., 2019). HIIT was defined as 77%–95% of HRmax, 64%–90% of VO_2_max, or 60%–89% of HRR, or an RPE ≥14 (Coates et al., 2023). The intervention group was required to include at least both a HIIT group or a MICT group, with a minimum intervention duration of more than 2 weeks, and detailed prescription information on frequency, mode, intensity, and volume. 3) Comparison: A no-training control group. 4) Outcomes: At least one outcome related to cardiometabolic health or body composition was required, including but not limited to heart rate, blood pressure, VO_2_max, muscular fitness, flexibility, fasting or postprandial glucose, blood lipids, insulin or lipid levels, BMI, and waist-to-hip ratio. In particular, VO2max was defined as the maximum rate of oxygen consumption during incremental exercise, reflecting cardiorespiratory fitness. VO_2_max data were included regardless of the specific assessment method used in the original studies, including laboratory-based tests (e.g., treadmill or cycle ergometer) and field-based tests (e.g., shuttle run). All VO_2_max outcomes were consistently categorized under cardiorespiratory fitness.5) Study design: Randomized controlled trials (RCT) and non-randomized trials. Exclusion criteria: qualitative studies, studies published in languages other than Chinese and English, systematic reviews/meta-analyses, study protocols, grey literature, abstracts, and commentaries were excluded.

### 2.3 Study selection

According to the PICOS criteria, one researcher (ZWH) independently used EndNote 20 software to remove duplicate records. Then, two researchers (ZWH and YMY) independently screened the remaining studies using Zotero seven based on titles and abstracts, following the predefined inclusion and exclusion criteria. For studies that could not be excluded based on titles and abstracts alone, the full texts were retrieved and assessed. Any discrepancies during the screening process were resolved by consultation with a third researcher (GY).

### 2.4 Data extraction and transformation

Data extraction was performed by the same two reviewers involved in the screening phase (ZWH and YMY) using a customized extraction form developed in Excel prior to full-text screening. The two reviewers independently extracted the following information: author and study details, participant characteristics, exercise intervention details, and outcome measurements. A third reviewer (GY) conducted an additional round of verification. In case of disagreement, a fourth independent reviewer (ZA) was consulted to reach consensus. If outcome data were missing or only presented in graphical format, the study authors were contacted to request the necessary information. If contact was unsuccessful and data were only available in figures, WebPlotDigitizer 4.1 (https://automeris.io/WebPlotDigitizer) was used to extract the relevant data. Studies for which data could not be successfully obtained were excluded from the final analysis.

### 2.5 Risk of bias and methodological quality

Risk of bias was independently assessed by two reviewers (ZWH and YMY). Any disagreements were resolved through discussion, and if consensus could not be reached, a third reviewer (GY) acted as an arbitrator. The assessment was conducted using the Cochrane Collaboration’s Risk of Bias 2 (RoB2) tool, which evaluates the following domains: random sequence generation, allocation concealment, blinding of participants and personnel, blinding of outcome assessment, completeness of outcome data, selective reporting, and other potential sources of bias (Sterne et al., 2019). In addition, the Physiotherapy Evidence Database (PEDro) scale (Maher et al., 2003) was used to supplement the RoB 2 assessment by providing a quantitative measure of methodological quality, particularly relevant to exercise intervention studies. The PEDro scale rates studies on a scale from 0 to 10; studies scoring ≥6 were considered high quality, those scoring 4–5 were considered moderate quality, and those scoring ≤3 were considered low quality (de Morton, 2009). Two reviewers (ZWH and GY) independently evaluated the included studies using the PEDro scale, and the scores were verified by a third reviewer (YMY).

### 2.6 Certainty of evidence

The strength of evidence from each study, combined with the quality rating, was used to inform the interpretation of findings. The certainty of evidence was assessed using the Grading of Recommendations Assessment, Development, and Evaluation (GRADE) approach, which classifies evidence as “high,” “moderate,” or “low” (Schünemann et al., 2019). The GRADE assessment was performed by one reviewer (ZWH) and verified by a second reviewer (YMY).

### 2.7 Statistical analysis

All statistical analyses in this study were performed using the “meta” and “metafor” packages in R software version 4.2.0 (Viechtbauer, 2010). The inverse variance method was applied using a random-effects model based on the DerSimonian–Laird approach, with tau^2^ and tau (and their confidence intervals) estimated using the Jackson method. Mean differences and standard deviations (SD) were extracted from each study to calculate pooled effect sizes, 95% confidence intervals (95% CI), and prediction intervals (DerSimonian and Laird, 1986). Given that outcome measures in this review typically involve different units of measurement, and in accordance with previous recommendations, standardized mean difference (SMD) was used as the preferred effect size metric (Nagashima et al., 2019). Considering that most of the included studies had small sample sizes, Hedges’ g was used as the effect size estimate. This metric is specifically corrected for small-sample bias and was calculated using the exact formula. The interpretation of Hedges’ g followed standard thresholds, with 0.2 indicating a small effect, 0.5 a moderate effect, and 0.8 a large effect (Cohen, 1988). Several statistics are available to assess heterogeneity (e.g., Cochrane’s Q, I^2^, tau^2^, and tau), but most methodological guidelines and textbooks recommend I^2^ as the primary indicator of heterogeneity. Therefore, I^2^ was reported as the main index, with the following interpretation: 0%–40% might not be important; 30%–60% represents moderate heterogeneity; 50%–90% indicates substantial heterogeneity; and 75%–100% indicates considerable heterogeneity (Higgins and Thompson, 2002). To explore potential sources of heterogeneity and moderators, subgroup analyses and meta-regression were conducted, focusing on two main dimensions: participant characteristics and exercise intervention protocols. Dichotomous variables (e.g., sex) were analyzed using subgroup analysis, while continuous variables (e.g., duration per session) were assessed using meta-regression (Hopkins, 2018). Specifically, participant-level moderators included BMI and age, while intervention-level moderators included exercise type, intensity, duration per session, frequency, and number of weeks. Publication bias was assessed using funnel plots (Peters et al., 2008) and Egger’s test (Egger et al., 1997), with p > 0.05 indicating no significant publication bias. A p-value <0.05 was considered statistically significant, and p-values between 0.05 and 0.10 were interpreted as indicating a trend toward significance.

## 3 Results

### 3.1 Search results

**FIGURE 1:**
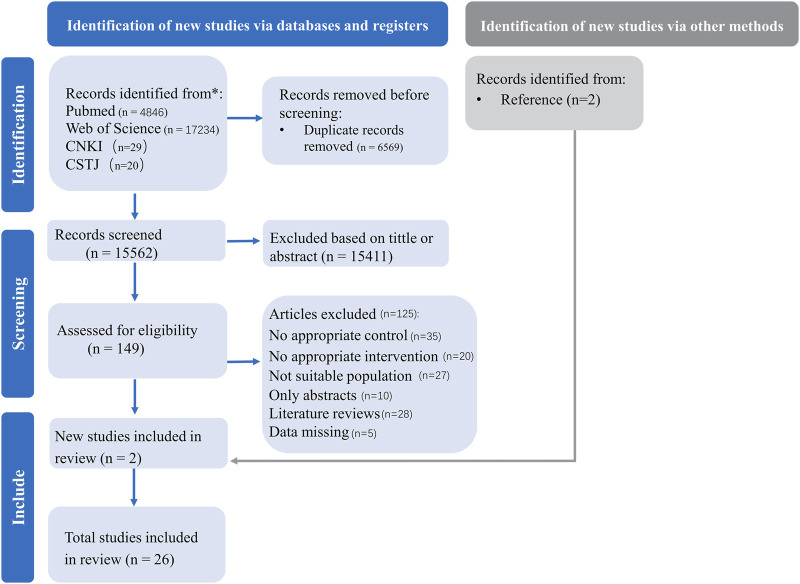
PRISMA flow diagram for included and excluded studies.

We systematically searched four databases and the initial search yielded 22,129 publications. Subsequently, we screened them, resulting in 26 studies (Cao et al., 2012; Koubaa, 2013; Lau et al., 2015; Archundia-Herrera et al., 2017; Shao and Fei, 2017; Chuensiri et al., 2018; Cvetkovic et al., 2018; Dias et al., 2018; Liang and Hao, 2018; Alizadeh and Safarzade, 2019a; 2019b; Miguet et al., 2020; Yuan, 2021; Cao et al., 2022b; Julian et al., 2022; Leite et al., 2022; Salus et al., 2022a; Salus et al., 2022b; Salus et al., 2023; Cao et al., 2022a; Cao et al., 2024; Abassi et al., 2023; D’Alleva et al., 2023; Tadiotto et al., 2023; Li et al., 2023; Su et al., 2024) for systematic review and meta-analysis (Figure 1).

### 3.2 Study characteristics

A total of 1,078 participants (765 males and 313 females; age range: 9–19 years) were included in the studies. The study populations consisted of overweight children (n = 7 studies), overweight adolescents (n = 5 studies), obese children (n = 4 studies), and obese adolescents (n = 10 studies). The outcomes assessed across the studies included BMI (n = 19), fat mass (n = 11), fat-free mass (n = 6), waist circumference (n = 11), weight (n = 18), cholesterol (n = 10), glucose (n = 6), insulin (n = 5), triglycerides (n = 7), VO_2_max (n = 12), diastolic blood pressure (DBP) (n = 9), systolic blood pressure (SBP) (n = 9), high-density lipoprotein (HDL) (n = 10), low-density lipoprotein (LDL) (n = 9), and HOMA-IR (n = 5). Additional study characteristics, including detailed descriptions of participants, study design, intervention protocols, and intervention duration and frequency, are provided in Table 1.

**TABLE 1:** The baseline characteristics of included studies.

Study	Population	Age (year)	Group	Group (n)	Protocol	Duration (weeks)	Frequency (days/week)
Su et al., 2024	Obese adolescents; n = 44 (0 female)	15 ± 1	HIIT	22	10 × 1-min running at (85%–95% HRpeak), interspersed with 10 × 2-min at (60%–70% HRpeak), with an RPE of 16–17	8	3
		14 ± 1	MICT	22	35-min running at (65%–75% HRpeak)		
Julian et al., 2022	Obese adolescents; n = 49 (29 female)	13.0 ± 1.1	HIIT	19	15 × 30s cycling at (85%–95% HRpeak)+30s active recovery (free but compulsory pedaling)+RT exercises	16	4
		13.0 ± 0.8	MICT	19	45-min running at (60% HRpeak)+RT exercises		
		13.2 ± 1.0	CON	11	No exercise		
Dias et al., 2018	Obese children; n = 99 (53 female)	12.4 ± 1.9	HIIT	33	4 × 4-min running at (85%–95% HRmax), interspersed with 3 × 3-min at (50%–70% HRmax)	12	3
		11.9 ± 2.4	MICT	32	44-min running at (60%–70% HRmax)		
		11.8 ± 2.4	CON	34	No exercise		
Miguet et al., 2020	Obese adolescents; n = 43 (31 female)	13.6 ± 1.5	HIIT	22	15 × 30s cycling at (85%–95% HRpeak)+30s active recovery (free but compulsory pedaling)	16	4
		13.6 ± 1.5	MICT	21	45-min running at (60% HRpeak)		
Leite et al., 2022	Obese children; n = 56 (0 female)	12.84 ± 1.87	HIIT	20	2 sets × 8 × 30s running/cycling at (100% MAS) Recovery: 60s; rest between sets:4min	12	3
		12.79 ± 1.56	MICT	20	45min indoor cycling (35%–55% HRR) 45min outdoor walking/running (35%–55% HRR)		
		12.58 ± 1.76	CON	16	No exercise		
Tadiotto et al., 2023	Overweight adolescents; n = 52 (26 female)	14.20 ± 1.96	HIIT	13	3 sets × 4 × 30s cycling at (80%–100% HRR), interspersed with 60s cycling for free	12	3
		14.20 ± 1.96	MICT	15	60-min running at (35%–75% HRR Increase every 4 weeks)		
		14.20 ± 1.96	CON	24	No exercise		
D’Alleva et al., 2023	Obese adolescents; n = 21 (0 female)	15.7 ± 1.7	HIIT + MICT	10	3 × 2-min running at (95% VO2peak), interspersed with 2-min at (50% VO2peak) 30-min running at (60%VO2peak)	3	28 ± 2
		16.2 ± 1.1	MICT	11	45-min running at (40%VO2peak)		
Archundia-Herrera et al., 2017	Obese adolescents; n = 30 (30 female)	16.13 ± 1.64	HIIT	15	6 × 1-min cycling at (85%–95% HRpeak), interspersed with 1-min at (free HRpeak)	1	1
		15.47 ± 1.73	MICT	15	40-min cycling at (65%HRpeak)		
Shao and Fei, 2017	Overweight adolescents; n = 35 (14 female)	18.1 ± 3.1	HIIT	19	6 × 3-min running at (80%–90% HRmax), interspersed with 7-min at (50%–60% HRmax)	9	5
		18.1 ± 3.1	MICT	16	Maintain intensity within the target heart rate range for swimming, running, and aerobic exercises		
Li et al., 2023	Overweight children; n = 60 (30 female)	11.0 ± 0.8	HIIT	20	3 sets × 8 × 15s running at (100%–120% MAS), interspersed with 15s running at (50%MAS); rest between sets:3 min	12	3
		11.0 ± 0.8	MICT	20	20–40 min running at (60–70%MAS) Every 4 weeks, the duration increases by 10 min and the intensity increases by 10%		
		11.0 ± 0.8	CON	20	No exercise		
Cao-a et al., 2022a	Overweight children; n = 60 (30 female)	11.2 ± 0.9	HIIT	20	3 sets × 8 × 15s running at (100%–120% MAS), interspersed with 15s running at (50%MAS); rest between sets:3 min	12	3
		10.9 ± 0.8	MICT	20	20–40 min running at (60–70%MAS) Every 4 weeks, the duration increases by 10 min and the intensity increases by 10%		
		10.9 ± 0.9	CON	20	No exercise		
Liang and Hao, 2018	Obese children; n = 56 (0 female)	n/a	HIIT	9	60s running at 100%speed, interspersed with 3min running at (50%speed)	12	3–6
		n/a	MICT	9	30–60 min running at (80%HRpeak) every 3 weeks, the duration increases by 10 min		
Abassi et al., 2023	Obese adolescents; n = 38 (38 female)	16.4 ± 1.2	HIIT	13	2 sets × 6–8 × 30s running at (100%–110% MAS), interspersed with 30s at (50% MAS); rest between sets:4min	12	3
		16.4 ± 1.2	MIIT	13	2 sets × 6–8 × 30s running at (60%–80% MAS), interspersed with 30s at (50% MAS); rest between sets:4min		
		16.4 ± 1.2	CON	12	No exercise		
Salus et al., 2022b	Obese adolescents; n = 28 (0 female)	13.1 ± 1.3	SIT	14	4–6 × 30s cycling at (100% MAS), interspersed with 4min at (free),Every 4 weeks, the duration increases by 1 times	12	3
		13.7 ± 1.6	CON	14	No exercise		
Salus et al., 2022a	Obese adolescents; n = 28 (0 female)	13.1 ± 1.3	SIT	14	4–6 × 30s cycling at (100% MAS), interspersed with 4min at (free),Every 4 weeks, the duration increases by 1 times	12	3
		13.7 ± 1.6	CON	14	No exercise		
Lau et al., 2015	Overweight children; n = 48 (12 female)	10.4 ± 0.9	HIIT	15	12 × 15s running at (120% MAS), interspersed with 15s recovery	6	3
		10.4 ± 0.9	LIIT	21	16 × 15s running at (100% MAS), interspersed with 15s recovery		
		10.4 ± 0.9	CON	12	No exercise		
Chuensiri et al., 2018	Overweight children; n = 37 (0 female)	11.0 ± 0.3	HIIT	11	8 × 2min cycling at (90% VO2peak), interspersed with 1min recovery	12	3
		11.1 ± 0.2	Supra-HIIT	15	8 × 20s cycling at (170% VO2peak), interspersed with 10s recovery		
		10.6 ± 0.3	CON	11	No exercise		
Cvetkovic et al., 2018	Overweight children; n = 35 (0 female)	11–13	HIIT	11	1–4weeks:3sets × 5 × 10s running at (100%MAS) interspersed with 10s recovery 5–8weeks:3sets × 8 × 15s running at (100%MAS) interspersed with 15s recovery 9–12weeks:3sets × 10 × 20s running at (100%MAS) interspersed with 20s recovery	12	3
		11–13	Football	10	A relative pitch area of 80 m^2^ per player and length to width aspect ratio of 2:1 Football game 4 × 8min playing interspersed with 2min recovery		
		11–13	CON	14	No exercise		
Alizadeh et al., 2019a	Overweight adolescents; n = 20 (0 female)	18.0 ± 1.5	HIIT	10	4–6 × 30s running at (90% HRmax), interspersed with 30s recovery	6	3
		18.0 ± 1.5	CON	10	No exercise		
Alizadeh et al., 2019b	Overweight adolescents; n = 20 (0 female)	18.0 ± 1.5	HIIT	10	4–6 × 30s running at (90% HRmax), interspersed with 30s recovery	6	3
		18.0 ± 1.5	CON	10	No exercise		
Cao et al., 2022b	Overweight children; n = 40 (20 female)	11.2 ± 0.7	HIIT	20	3 sets × 8 × 15s running at (80%–90% HRmax), interspersed with 15s running at (40%HRmax); rest between sets:3 min	12	3
		10.9 ± 0.4	CON	20	No exercise		
Salus et al., 2023	Obese children; n = 28 (0 female)	13.1 ± 0.4	SIT	14	4–6 × 30s cycling at (100% MAS), interspersed with 4min at (free),Every 4 weeks, the duration increases by 1 times	12	3
		13.7 ± 0.4	CON	14	No exercise		
Cao et al., 2024	Overweight children; n = 42 (0 female)	12.4 ± 0.4	HIIT	14	3 sets × 8 × 15s running at (100% MAS), interspersed with 15s recovery; rest between sets:3 min	12	3
		12.1 ± 0.6	Miit	14	3 sets × 8 × 15s running at (80% MAS), interspersed with 15s recovery; rest between sets:3 min		
		12.4 ± 0.5	CON	14	No exercise		
Yuan, 2021	Overweight adolescents; n = 40 (0 female)	16.1 ± 1.2	HIIT	20	1–3weeks:2sets × 5 × 30s cycling at (100%MAP) interspersed with 30s cycling at (50%MAP) 4–6weeks:3sets × 6 × 30s cycling at (100%MAP) interspersed with 30s cycling at (50%MAP) 7–9weeks:4sets × 7 × 30s cycling at (100%MAP) interspersed with 30s cycling at (50%MAP) 10–12weeks:5sets × 8 × 30s cycling at (100%MAP) interspersed with 30s cycling at (50%MAP)	12	3
		15.9 ± 1.2	CON	20	No exercise		
Cao et al., 2012	Obese adolescents; n = 40 (0 female)	13–15	MICT	20	4 × 4min running at (90–95%HRmax) and 3min cycling at (70%HRmax)	8	2
		13–15	CON	20	No exercise		
Koubaa, 2013	Obese adolescents; n = 29 (0 female)	12.9 ± 0.5	HIIT	14	Every time 2 min running at (80%VO_2_max) interspersed with1min recovery	12	3
		13 ± 0.8	MICT	15	at 60% of vVO_2_max (first 4 weeks), 65% of VO_2_max (second 4 weeks) and 70% of vVO2 max (3rd 4 weeks) Others are not specified		

CON, control group; HIIT, high-intensity interval training; MICT, moderate-intensity continuous training; MAS, maximum aerobic speed; MAP, maximal aerobic power; HRmax, Maximum Heart Rate; VO_2_max, Maximal Oxygen Uptake; HRpeak, Peak Heart Rate; RPE, rating of perceived exertion; HRR, heart rate reserve; VO2peak, Peak Oxygen Uptake.

### 3.3 Methodological quality of included studies

The obtained PEDro scores ranged from moderate to high quality (4–9) for the systematic review and meta-analysis, with an average score of 5.88 ± 1.24. Among the included studies, 22 (84.6%) were rated as moderate quality (scores of 5–7), 2 studies (7.7%) were rated as high quality (≥8), and 2 studies (7.7%) were considered low quality (≤4). The most frequently observed scores were 5 and 6, accounting for 34.6% of all studies. Table 2 provides a detailed summary of the methodological quality assessment, including individual PEDro scores for each study.

**TABLE 2:** Methodological quality assessment (PEDro).

Author, year	D1	D2	D3	D4	D5	D6	D7	D8	D9	D10	D11	Total
Julian et al., 2022	Y	1	0	1	0	0	0	0	1	1	1	5
Su et al., 2024	Y	1	1	1	0	1	1	1	1	1	1	9
Dias et al., 2018	Y	1	1	0	0	0	0	0	1	1	1	5
Miguet et al., 2020	Y	1	0	1	0	0	0	1	1	1	1	6
Leite et al., 2022	Y	0	0	1	0	0	0	1	1	1	1	5
Tadiotto et al., 2023	Y	0	0	1	0	0	0	1	1	1	1	5
D’Alleva et al., 2023	Y	1	1	1	0	0	0	1	1	1	1	7
Archundia-Herrera et al., 2017	Y	1	0	1	0	0	0	1	1	1	1	6
Shao and Fei, 2017	Y	0	0	1	0	0	0	1	1	1	1	5
Li et al., 2023	Y	1	0	1	0	0	0	0	1	1	1	5
Cao et al., 2022a	Y	1	1	1	0	0	0	1	1	1	1	7
Liang and Hao, 2018	Y	1	0	1	0	0	0	1	1	1	1	6
Alizadeh and afarzade, 2019a	Y	1	0	1	0	0	0	1	1	1	1	6
Salus et al., 2022a	Y	0	0	1	0	0	0	1	1	1	1	5
Salus et al., 2022b	Y	0	0	1	0	0	0	0	1	1	1	4
Lau et al., 2015	Y	0	0	1	0	0	0	0	1	1	1	4
Chuensiri et al., 2018	Y	1	0	1	0	0	0	0	1	1	1	5
Abassi et al., 2023	Y	1	0	1	0	0	0	1	1	1	1	6
Cvetkovic et al., 2018	Y	1	0	1	0	1	1	0	1	1	1	7
Alizadeh et al., 2019b	Y	1	0	1	0	0	0	1	1	1	1	6
Cao et al., 2022b	Y	1	1	1	0	1	1	1	1	1	1	9
Salus et al., 2023	Y	1	0	1	0	0	0	0	1	1	1	5
Cao et al., 2012	Y	1	0	1	0	0	0	1	1	1	1	6
Koubaa, 2013	Y	1	0	1	0	0	0	1	1	1	1	6
Cao et al., 2024	Y	1	1	1	0	0	0	1	1	1	1	7
Yuan, 2021	Y	1	0	1	0	0	0	1	1	1	1	6

Studies scoring ≥6 is considered high quality, those scoring 4–5 are considered moderate quality, and those scoring ≤3 are considered low quality.

1. Eligibility criteria were specified (not included in the total score).

2. Subjects were randomly allocated to groups (in a crossover study, subjects were randomly allocated an order in which treatments were received).

3. Allocation was concealed.

4. The groups were similar at baseline regarding the most important prognostic indicators.

5. There was blinding of all subjects.

6. There was blinding of all therapists who administered the therapy.

7. There was blinding of all assessors who measured at least one key outcome.

8. Measures of at least one key outcome were obtained from more than 85% of the subjects initially allocated to groups.

9. All subjects for whom outcome measures were available received the treatment or control condition as allocated or, where this was not the case, data for at least one key outcome was analyzed by “intention to treat”.

10. The results of between-group statistical comparisons are reported for at least one key outcome.

11. The study provides both point measures and measures of variability for at least one key outcome.

### 3.4 Effects of HIIT on health outcomes compared with no-training

**FIGURE 2:**
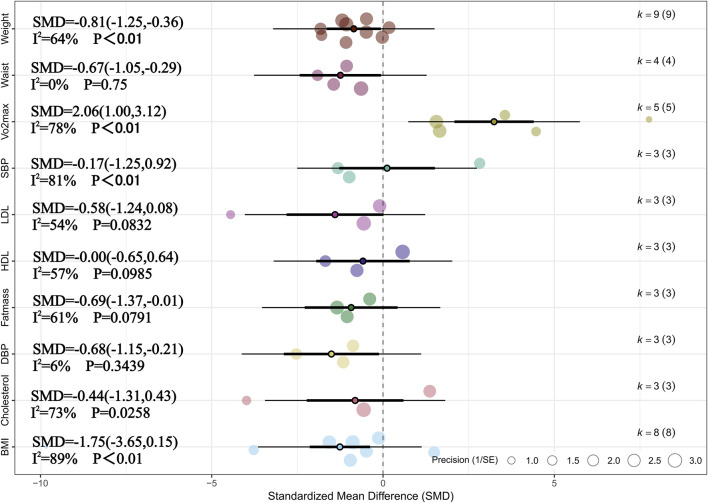
Summary of the impact of HIIT vs. CON on health outcomes.

Meta-analysis of three studies revealed that, compared with no training, HIIT had a significant overall effect on fat mass in children and adolescents with overweight or obesity (SMD = −0.69, 95% CI [–1.37, −0.01], p < 0.05) (Cvetkovic et al., 2018; Cao et al., 2022b; Salus et al., 2022a). Moderate heterogeneity was observed across studies (I^2^ = 61%, Prediction interval [-3.19, 1.80], p > 0.05), indicating relatively consistent findings among individual studies. In addition, sensitivity analysis confirmed that the effect of HIIT on reducing fat mass remained robust (SMD = −0.69, 95% CI [–1.37, −0.01], p < 0.05). Meta-analysis of four studies showed that HIIT had a significant overall effect on waistline (SMD = −0.67, 95% CI [–1.05, −0.29], p < 0.01) (Chuensiri et al., 2018; Yuan, 2021; Salus et al., 2022a; Abassi et al., 2023). Heterogeneity across studies was low (I^2^ = 0%, p > 0.05), suggesting high consistency. Sensitivity analysis also indicated that the effect of HIIT on waistline reduction was robust (SMD = −0.67, 95% CI [–1.05, −0.29], p < 0.01). Meta-analysis of nine studies demonstrated a significant overall effect of HIIT on weight reduction (SMD = −0.81, 95% CI [–1.25, −0.36], p < 0.05), with moderate heterogeneity observed (I^2^ = 64%, Prediction interval [-2.16, 0.54], p < 0.05) (Lau et al., 2015; Chuensiri et al., 2018; Cvetkovic et al., 2018; Alizadeh and Safarzade, 2019b; Yuan, 2021; Cao et al., 2022a; Salus et al., 2022a; Abassi et al., 2023; Cao et al., 2024). Sensitivity analysis showed that the result was relatively robust (SMD = −0.81, 95% CI [–1.25, −0.36], p < 0.05). Meta-analysis of five studies indicated that HIIT significantly improved VO_2_max (SMD = 2.06, 95% CI [1.00, 3.12], p < 0.01), with substantial heterogeneity across studies (I^2^ = 78%, Prediction interval [-1.37, 5.48], p < 0.01) (Chuensiri et al., 2018; Yuan, 2021; Cao et al., 2022b; Salus et al., 2022a; Cao et al., 2024). Sensitivity analysis supported the robustness of the result (SMD = 1.26, 95% CI [0.61, 1.90], p < 0.05). Meta-analysis of three studies showed that HIIT had a significant effect on DBP (SMD = −0.68, 95% CI [–1.15, −0.21], p < 0.05), with low heterogeneity observed (I^2^ = 6%, p < 0.05) (Chuensiri et al., 2018; Cvetkovic et al., 2018; Salus et al., 2022b). Sensitivity analysis confirmed that this finding was robust (SMD = −0.68, 95% CI [–1.15, −0.21], p < 0.05). In addition, no statistically significant effects of HIIT were observed on BMI, cholesterol, SBP, HDL, or LDL (p > 0.05 for all). For more detailed information, see Figure 2 and Supplementary Figure 1.

The following are the results of subgroup analyses examining the effects of HIIT on health outcomes compared with no training. Due to the limited number of eligible studies (n < 5), subgroup analyses were only conducted for weight, BMI, and VO_2_max. This analysis aimed to explore whether factors such as overweight or obesity status, age differences, sex, intervention modality, and intervention duration influenced the effects of HIIT. The results indicated that overweight or obesity status had a statistically significant impact on improvements in VO_2_max (p < 0.05). Although slight differences in effect sizes were observed across other subgroups, none of these differences reached statistical significance (p_b_> 0.05). It should be noted that some subgroups, particularly those involving obesity status, included only two to three studies. This limited number may reduce the statistical power and affect the stability of the estimates. Therefore, these results should be interpreted with caution and considered exploratory. For detailed information, see Table 3 and Supplementary Table 4.

**TABLE 3:** BMI-based subgroup analysis of health outcomes in HIIT vs. No-training.

Outcomes	Subgroup	K	Hedges' *g*	95% CI	*p*_v_	*I* ^2^	*p*_b_
Weight	Obesity	2	−0.59	[-1.51, 0.34]	0.21	64%	0.601
	Overweight	7	−0.87	[-1.41, −0.34]	<0.01	68%	
VO2max	Obesity	1	0.95	[0.16, 1.73]	n/a	n/a	0.042
	Overweight	4	2.31	[1.31, 3.31]	<0.01	78%	

K, the total number of effects included in the pooled effect size; Hedges' g, the effect size indicators used in the pooled; p_v_, overall pooled effect; p_b_, between subgroups differences; 95%CI, 95% confidence interval; I^2^, quantitative indicators of heterogeneity; VO2max, Maximal Oxygen Uptake.

### 3.5 Effects of MICT on health outcomes compared with no-training

**FIGURE 3:**
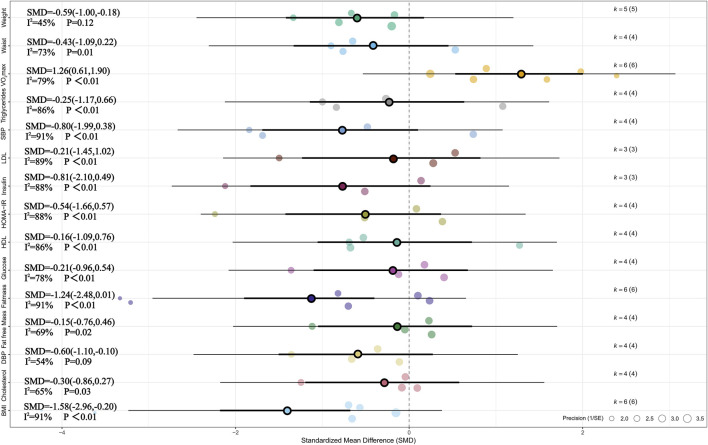
Summary of the impact of MICT vs. CON on health outcomes.

Meta-analysis of six studies showed that, compared with the non-exercise group, MICT had a significant effect on BMI (SMD = −1.58, 95% CI [–2.96, −0.20], p < 0.05) (Cao et al., 2012; Dias et al., 2018; Julian et al., 2022; Leite et al., 2022; Cao et al., 2022a; Li et al., 2023). Substantial heterogeneity was observed among studies (I^2^ = 91%, Prediction interval [-6.23, 3.07], p < 0.01), and sensitivity analysis confirmed that the result was highly robust (SMD = −1.58, 95% CI [–2.96, −0.20], p < 0.05). Meta-analysis of five studies indicated a significant effect of MICT on weight (SMD = −0.59, 95% CI [–1.00, −0.18], p < 0.01), with moderate heterogeneity across studies (I^2^ = 45%, p > 0.05) (Cao et al., 2012; Dias et al., 2018; Julian et al., 2022; Leite et al., 2022; Li et al., 2023). Sensitivity analysis confirmed the robustness of this finding (SMD = −0.59, 95% CI [–1.00, −0.18], p < 0.01). Meta-analysis of six studies found a significant effect of MICT on VO_2_max (SMD = 1.26, 95% CI [0.61, 1.90], p < 0.01), with substantial heterogeneity (I^2^ = 79%, Prediction interval [-0.77, 3.28],p < 0.05), and sensitivity analysis demonstrated that the result was highly robust (SMD = 1.26, 95% CI [0.61, 1.90], p < 0.01) (Cao et al., 2012; Dias et al., 2018; Leite et al., 2022; Cao et al., 2022b; Tadiotto et al., 2023; Li et al., 2023). Additionally, meta-analysis of four studies indicated a significant effect of MICT on DBP (SMD = −0.60, 95% CI [–1.10, −0.10], p < 0.05), with moderate heterogeneity among studies (I^2^ = 54%, Prediction interval [-2.04, 0.84], p > 0.05) (Cao et al., 2012; Leite et al., 2022; Cao et al., 2022a; Tadiotto et al., 2023). Sensitivity analysis confirmed the robustness of this finding as well (SMD = −0.60, 95% CI [–1.10, −0.10], p < 0.05). For more details, see Figure 3 and Supplementary Figure 2.

Subgroup analysis of the effects of MICT on health outcomes compared with no exercise showed that, among the potential influencing factors including BMI, age, gender, and training modes, only overweight or obesity status in children and adolescents had a statistically significant impact on weight change (Hedges’ g = −0.59). No statistically significant differences were found among the other subgroups. Similarly, it should be noted that several subgroups—particularly those based on BMI classification, age group, and gender—included only two to three studies. This limited number of included studies may affect the stability and reliability of the estimated effects. Therefore, these subgroup findings should be considered exploratory in nature and interpreted with caution. For detailed information, see Table 4 and Supplementary Table 5.

**TABLE 4:** BMI-based subgroup analysis of health outcomes in MICT vs. No-training.

Outcomes	Subgroup	K	Hedges' *g*	95% CI	*p*_v_	*I* ^2^	*p*_b_
Weight	Obesity	3	−0.30	[-0.67, 0.07]	0.11	0%	0.024
	Overweight	2	−1.02	[-1.53, −0.52]	<0.01	2%	
VO2max	Obesity	3	1.01	[0.03, 1.99]	0.04	84%	0.453
	Overweight	3	1.53	[0.60, 2.45]	<0.01	75%	
Fat mass	Obesity	3	−0.45	[-1.03, 0.13]	0.13	52%	0.187
	Overweight	3	−2.06	[-4.39, 0.26]	0.08	96%	

K, the total number of effects included in the pooled effect size; Hedges' g, the effect size indicators used in the pooled; p_v_, overall pooled effect; p_b_, between subgroups differences; 95%CI, 95% confidence interval; I^2^, quantitative indicators of heterogeneity; VO2max, Maximal Oxygen Uptake.

### 3.6 Effects of HIIT on health outcomes compared with MICT

**FIGURE 4:**
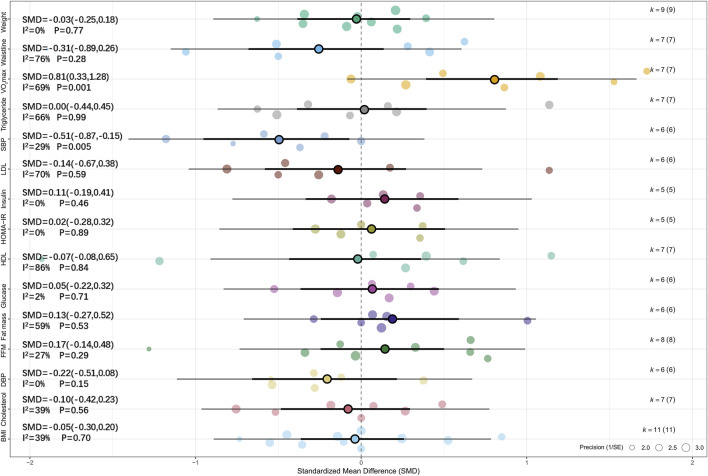
Summary of the impact of HIIT vs. MICT on health outcomes.

Meta-analysis of six studies showed that, compared with MICT, HIIT had a significant effect on SBP (SMD = −0.51, 95% CI [–0.87, −0.15], p < 0.01) (Koubaa, 2013; Liang and Hao, 2018; Leite et al., 2022; Cao et al., 2022b; Tadiotto et al., 2023; Li et al., 2023). Low heterogeneity was observed across studies (I^2^ = 29%, p > 0.05), and sensitivity analysis indicated that the result was relatively robust (SMD = −0.51, 95% CI [–0.88, −0.14], p < 0.05). Meta-analysis of seven studies revealed that, compared with MICT, HIIT had a significant effect on VO_2_max (SMD = 0.81, 95% CI [0.33, 1.28], p = 0.01, I^2^ = 69%, Prediction interval [-0.63, 2.24], p < 0.05), and sensitivity analysis confirmed the robustness of the result (SMD = 0.81, 95% CI [0.32, 1.29], p = 0.001) (Koubaa, 2013; Dias et al., 2018; Leite et al., 2022; Cao et al., 2022a; Tadiotto et al., 2023; Li et al., 2023; Su et al., 2024). For more details, see Figure 4 and Supplementary Figure S3.

The following are the results of subgroup analyses comparing the effects of HIIT *versus* MICT on health outcomes. We found that overweight or obesity status and sex were significant moderators of the effect of HIIT on SBP in children and adolescents (Hedges’ g = −0.51). In addition, sex was also a significant moderator of the effect of HIIT on HDL (Hedges’ g = −0.07) and LDL (Hedges’ g = −0.14) in children and adolescents with overweight or obesity. Age (≤12 years) significantly moderated the effect of HIIT on HDL (Hedges’ g = −0.07), while training frequency significantly moderated the effects of HIIT on BMI (Hedges’ g = −0.05) and fat mass (Hedges’ g = −0.13) in this population. Furthermore, training mode was found to significantly moderate the effect of HIIT on VO_2_max (Hedges’ g = 0.81). No statistically significant differences were found between HIIT and MICT conditions across the remaining subgroups (p > 0.05). It is worth noting that several subgroup analyses involving glycolipid metabolism indicators (such as glucose, insulin, and cholesterol) included only two to three studies. The limited sample size may reduce the robustness and statistical power of the effect estimates; therefore, these results should be interpreted with caution. For detailed results, see Tables 5–9 and Supplementary Table 3.

**TABLE 5:** BMI-based subgroup analysis of health outcomes in HIIT vs. MICT.

Outcomes	Subgroup	K	Hedges' *g*	95% CI	*p*_v_	*I* ^2^	*p*_b_
Fat mass	Obesity	5	−0.05	[-0.59, 0.49]	0.86	66%	0.229
	Overweight	3	0.41	[-0.10, 0.91]	0.12	32%	
Free fat mass	Obesity	4	0.04	[-0.27, 0.35]	0.78	0%	0.382
	Overweight	2	0.50	[-0.48, 1.49]	0.32	72%	
Waistline	Obesity	3	−0.19	[-1.45, 1.06]	0.76	86%	0.720
	Overweight	4	−0.44	[-0.97, 0.09]	0.10	56%	
Weight	Obesity	7	−0.05	[-0.30, 0.20]	0.70	1%	0.785
	Overweight	2	0.02	[-0.44, 0.49]	0.92	0%	
Cholesterol	Obesity	4	−0.14	[-0.65, 0.37]	0.59	56%	0.722
	Overweight	3	−0.02	[-0.45, 0.42]	0.93	21%	
Glucose	Obesity	3	0.11	[-0.26, 0.48]	0.55	0%	0.672
	Overweight	3	−0.02	[-0.51, 0.47]	0.94	35%	
Insulin	Obesity	2	0.06	[-0.47, 0.58]	0.83	18%	0.760
	Overweight	3	0.16	[-0.23, 0.55]	0.43	0%	
Triglyceride	Obesity	4	0.17	[-0.54, 0.89]	0.63	77%	0.387
	Overweight	3	−0.21	[-0.69, 0.28]	0.41	34%	
VO2max	Obesity	4	0.67	[-0.01, 1.34]	0.05	76%	0.485
	Overweight	3	1.01	[0.32, 1.71]	<0.01	59%	
DBP	Obesity	3	−0.45	[-0.88, −0.03]	0.03	0%	0.131
	Overweight	3	0	[-0.41, 0.41]	0.99	0%	
SBP	Obesity	3	−0.88	[-1.33, −0.44]	<0.01	0%	0.024
	Overweight	3	−0.19	[-0.60, 0.23]	0.377	0%	
HDL	Obesity	4	−0.51	[-1.66, 0.63]	0.38	90%	0.134
	Overweight	3	0.48	[-0.14, 1.10]	0.13	57%	
LDL	Obesity	3	0.14	[-0.96, 1.25]	0.80	86%	0.373
	Overweight	3	−0.39	[-0.78, 0.01]	0.05	0%	
HOMA-IR	Obesity	3	−0.05	[-0.42, 0.32]	0.78	0%	0.502
	Overweight	2	0.16	[-0.34, 0.67]	0.53	0%	

K, the total number of effects included in the pooled effect size; Hedges' g, the effect size indicators used in the pooled; p_v_, overall pooled effect; p_b_, between subgroups differences; 95%CI, 95% confidence interval; I^2^, quantitative indicators of heterogeneity; VO2max, Maximal Oxygen Uptake; DBP, diastolic blood pressure; SBP, systolic blood pressure; HDL, High-Density Lipoprotein; LDL, Low-Density Lipoprotein; HOMA-IR, homeostatic model assessment of insulin resistance.

**TABLE 6:** Age-based subgroup analysis of health outcomes in HIIT vs. MICT.

Outcomes	Subgroup	K	Hedges' *g*	95% CI	*p*_v_	*I* ^2^	*p*_b_
BMI	>12 years	7	0	[-0.36, 0.35]	0.99	52%	0.680
	≤12 years	4	−0.11	[-0.51, 0.28]	0.57	19%	
Fat mass	>12 years	4	0.14	[-0.33, 0.61]	0.55	46%	0.870
	≤12 years	4	0.07	[-0.65, 0.80]	0.84	74%	
Free fat mass	>12 years	4	0.01	[-0.32, 0.34]	0.23	0%	0.260
	≤12 years	2	0.53	[-0.33, 1.39]	0.96	71%	
Waistline	>12 years	4	0	[-0.58, 0.58]	0.99	65%	0.220
	≤12 years	3	−0.83	[-2.03, 0.37]	0.18	84%	
Weight	>12 years	6	−0.04	[-0.30, 0.22]	0.76	0%	0.950
	≤12 years	3	−0.03	[-0.45, 0.40]	0.90	8%	
Cholesterol	>12 years	5	0.02	[-0.31, 0.35]	0.90	12%	0.400
	≤12 years	2	−0.35	[-1.17, 0.46]	0.39	68%	
Glucose	>12 years	4	0.25	[-0.09, 0.59]	0.14	0%	0.053
	≤12 years	2	−0.30	[-0.75, 0.15]	0.19	0%	
Insulin	>12 years	4	0,13	[-0.20, 0.47]	0.44	0%	0.810
	≤12 years	1	0.11	[-0.19, 0.41]	n/a	n/a	
Triglyceride	>12 years	5	0.18	[-0.37, 0.73]	0.52	68%	0.096
	≤12 years	2	−0.43	[-0.88, 0.03]	0.06	0%	
VO2max	>12 years	4	0.51	[-0.10, 1.12]	0.10	68%	0.082
	≤12 years	3	1.19	[0.72, 1.66]	<0.01	20%	
DBP	>12 years	3	−0.34	[-0.74, 0.06]	0.09	0%	0.470
	≤12 years	3	−0.09	[-0.63, 0.44]	0.73	31%	
SBP	>12 years	3	−0.74	[-1.22, −0.25]	<0.01	26%	0.140
	≤12 years	3	−0.24	[-0.68, 0.19]	0.28	0%	
HDL	>12 years	5	−0.42	[-1.30, 0.47]	0.36	86%	0.049
	≤12 years	2	0.74	[0, 1.47]	0.05	58%	
LDL	>12 years	4	0.12	[-0.54, 0.78]	0.72	71%	0.060
	≤12 years	2	−0.66	[-1.12, −0.19]	<0.01	0%	
HOMA-IR	>12 years	3	0.16	[-0.24, 0.56]	0.42	0%	0.295
	≤12 years	2	−0.16	[-0.61, 0.29]	0.49	0%	

K, the total number of effects included in the pooled effect size; Hedges' g, he effect size indicators used in the pooled; p_v_, overall pooled effect; p_b_, between subgroups differences; 95%CI, 95% confidence interval; I^2^, quantitative indicators of heterogeneity; BMI, Body Mass Index; VO2max, Maximal Oxygen Uptake; DBP, diastolic blood pressure; SBP, systolic blood pressure; HDL, High-Density Lipoprotein; LDL, Low-Density Lipoprotein; HOMA-IR, homeostatic model assessment of insulin resistance.

**TABLE 7:** Gender-based subgroup analysis of health outcomes in HIIT vs. MICT.

Outcomes	Subgroup	K	Hedges' *g*	95% CI	*p*_v_	*I* ^2^	*p*_b_
BMI	Male	4	0.16	[-0.45, 0.78]	0.60	65%	0.334
	Mixed	7	−0.16	[-0.41, 0.08]	0.19	0%	
Fat mass	Male	3	0	[-1.02, 1.03]	0.99	80%	0.804
	Mixed	5	0.14	[-0.26, 0.54]	0.48	43%	
Free fat mass	Male	2	−0.08	[-0.55, 0.39]	0.74	0%	0.241
	Mixed	4	0.30	[-0.12, 0.71]	0.16	37%	
Waistline	Male	3	−0.19	[-1.45, 1.06]	0.76	86%	0.720
	Mixed	4	−0.44	[-0.97, 0.09]	0.10	56%	
Weight	Male	4	0.04	[-0.32, 0.40]	0.84	8%	0.610
	Mixed	5	−0.08	[-0.36, 0.20]	0.57	0%	
Cholesterol	Male	2	−0.11	[-0.58, 0.37]	0.66	0%	0.264
	Female	1	0.49	[-0.24, 1.22]	n/a	n/a	
	Mixed	4	−0.23	[-0.72, 0.26]	0.36	54%	
Glucose	Male	1	0.44	[-0.19, 1.07]	n/a	n/a	0.395
	Female	1	0.07	[-0.65, 0.78]	n/a	n/a	
	Mixed	4	−0.06	[-0.39, 0.28]	0.75	6%	
Insulin	Male	1	−0.18	[-0.80, 0.44]	n/a	n/a	0.513
	Female	1	0.36	[-0.37, 1.08]	n/a	n/a	
	Mixed	3	0.16	[-0.23, 0.55]	0.43	0%	
Triglyceride	Male	2	0.54	[-0.64, 1.72]	0.37	82%	0.276
	Female	1	0.16	[-0.56, 0.88]	n/a	n/a	
	Mixed	4	−0.29	[-0.66, 0.08]	0.13	21%	
VO2max	Male	3	0.53	[-0.31, 1.37]	0.21	78%	0.312
	Mixed	4	1.03	[0.56, 1.50]	<0.01	39%	
DBP	Male	3	−0.45	[-0.88, −0.03]	0.03	0%	0.132
	Mixed	3	0	[-0.41, 0.41]	0.99	0%	
SBP	Male	3	−0.88	[-1.33, −0.44]	<0.01	0%	0.020
	Mixed	3	−0.19	[-0.60, 0.23]	0.38	0%	
HDL	Male	2	−1.51	[-2.19, −0.82]	<0.01	34%	<0.01
	Female	1	0.62	[-0.12, 1.35]	n/a	n/a	
	Mixed	4	0.45	[0.03, 0.88]	0.03	37%	
LDL	Male	1	1.14	[0.34, 1.93]	n/a	n/a	<0.01
	Female	1	0.17	[-0.54, 0.89]	n/a	n/a	
	Mixed	4	−0.51	[-0.84, −0.18]	<0.01	0%	
HOMA-IR	Male	1	−0.12	[-0.74, 0.50]	n/a	n/a	0.561
	Female	1	0.37	[-0.35, 1.09]	n/a	n/a	
	Mixed	3	−0.02	[-0.41, 0.36]	0.91	0%	

K, the total number of effects included in the pooled effect size; Hedges' g, the effect size indicators used in the pooled; p_v_, overall pooled effect; p_b_, between subgroups differences; 95%CI, 95% confidence interval; I^2^, quantitative indicators of heterogeneity; BMI, Body Mass Index; VO2max, Maximal Oxygen Uptake; DBP, diastolic blood pressure; SBP, systolic blood pressure; HDL, High-Density Lipoprotein; LDL, Low-Density Lipoprotein; HOMA-IR, homeostatic model assessment of insulin resistance.

**TABLE 8:** Frequency-based subgroup analysis of health outcomes in HIIT vs. MICT.

Outcomes	Subgroup	K	Hedges' *g*	95% CI	*p*_v_	*I* ^2^	*p*_b_
BMI	>3 times	4	−0.38	[-0.72, −0.04]	0.03	0%	0.023
	≤3 times	7	0.14	[-0.15, 0.42]	0.35	28%	
Fat mass	>3 times	2	−0.72	[-1.62, 0.19]	0.12	56%	0.029
	≤3 times	6	0.34	[0.04, 0.64]	0.03	16%	
Free fat mass	>3 times	1	0.16	[-0.44, 0.75]	n/a	n/a	0.957
	≤3 times	5	0.18	[-0.22, 0.57]	0.38	42%	
Waistline	>3 times	2	−1.11	[-2.46, 0.23]	0.10	76%	0.153
	≤3 times	5	−0.04	[-0.65, 0.57]	0.90	73%	
Weight	>3 times	4	−0.26	[-0.60, 0.07]	0.13	0%	0.079
	≤3 times	5	0.13	[-0.15, 0.42]	0.36	0%	
Glucose	>3 times	1	0.17	[-0.45, 0.79]	n/a	n/a	0.690
	≤3 times	5	0.03	[-0.31, 0.36]	0.88	19%	
Triglyceride	>3 times	1	0.21	[-0.41, 0.84]	n/a	n/a	0.548
	≤3 times	6	−0.04	[-0.56, 0.49]	0.89	71%	
DBP	>3 times	1	−0.54	[-1.49, 0.40]	n/a	n/a	0.475
	≤3 times	5	−0.18	[-0.49, 0.13]	0.25	0%	
SBP	>3 times	1	−0.77	[-1.74, 0.19]	n/a	n/a	0.577
	≤3 times	5	−0.47	[-0.89, −0.06]	0.02	40%	
HDL	>3 times	1	0.27	[-0.36, 0.89]	n/a	n/a	0.459
	≤3 times	6	−0.14	[-1.00, 0.73]	0.76	88%	
LDL	>3 times	1	−0.26	[-0.88, 0.37]	n/a	n/a	0.756
	≤3 times	5	−0.11	[-0.77, 0.54]	0.73	76%	

K, the total number of effects included in the pooled effect size; Hedges' g, the effect size indicators used in the pooled; p_v_, overall pooled effect; p_b_, between subgroups differences; 95%CI, 95% confidence interval; I^2^, quantitative indicators of heterogeneity; BMI, body mass index; DBP, diastolic blood pressure; SBP, systolic blood pressure; HDL, High-Density Lipoprotein; LDL, Low-Density Lipoprotein.

**TABLE 9:** Training Modes-based subgroup analysis of health outcomes in HIIT vs. MICT.

Outcomes	Subgroup	K	Hedges' *g*	95% CI	*p*_v_	*I* ^2^	*p*_b_
BMI	Running	7	0.01	[-0.30, 0.32]	0.95	33%	0.599
	Cycling	4	−0.15	[-0.64, 0.35]	0.56	56%	
Fat mass	Running	5	0.22	[-0.39, 0.82]	0.49	71%	0.504
	Cycling	3	−0.03	[-0.44, 0.37]	0.87	10%	
Free fat mass	Running	3	0.27	[-0.42, 0.97]	0.44	69%	0.640
	Cycling	3	0.08	[-0.29, 0.46]	0.66	0%	
Waistline	Running	5	−0.45	[-1.22, 0.32]	0.25	80%	0.474
	Cycling	2	−0.02	[-0.91, 0.88]	0.97	70%	
Weight	Running	6	0.04	[-0.23, 0.31]	0.77	0%	0.395
	Cycling	3	−0.16	[-0.53, 0.21]	0.40	5%	
Cholesterol	Running	4	−0.11	[-0.58, 0.35]	0.63	50%	0.906
	Cycling	3	−0.07	[-0.62, 0.48]	0.80	47%	
Glucose	Running	3	−0.14	[-0.52, 0.23]	0.46	6%	0.129
	Cycling	3	0.28	[-0.12, 0.68]	0.164	0%	
Insulin	Running	2	0.09	[-0.37, 0.55]	0.70	0%	0.896
	Cycling	3	0.13	[-0.27, 0.53]	0.52	0%	
Triglyceride	Running	4	−0.18	[-0.50, 0.15]	0.29	0%	0.442
	Cycling	3	0.24	[-0.77, 1.24]	0.64	83%	
VO2max	Running	5	1.05	[0.53, 1.57]	<0.01	62%	0.021
	Cycling	2	0.17	[-0.37, 0.71]	0.53	19%	
DBP	Running	4	−0.13	[-0.52, 0.26]	0.51	5%	0.461
	Cycling	2	−0.36	[-0.84, 0.12]	0.14	0%	
SBP	Running	4	−0.33	[-0.71, 0.05]	0.09	0%	0.305
	Cycling	2	−0.79	[-1.58, 0.00]	0.05	59%	
HDL	Running	4	0	[-1.07, 1.08]	0.99	89%	0.811
	Cycling	3	−0.18	[-1.28, 0.91]	0.74	86%	
LDL	Running	4	−0.13	[-0.89, 0.64]	0.75	80%	0.958
	Cycling	2	−0.15	[-0.81, 0.51]	0.65	38%	
HOMA-IR	Running	2	−0.16	[-0.61, 0.29]	0.49	0%	0.295
	Cycling	3	0.16	[-0.24, 0.56]	0.42	0%	

K, the total number of effects included in the pooled effect size; Hedges' g, the effect size indicators used in the pooled; p_v_, overall pooled effect; p_b_, between subgroups differences; 95%CI, 95% confidence interval; I^2^, quantitative indicators of heterogeneity; BMI, Body Mass Index; VO2max, Maximal Oxygen Uptake; DBP, diastolic blood pressure; SBP, systolic blood pressure; HDL, High-Density Lipoprotein; LDL, Low-Density Lipoprotein; HOMA-IR, homeostatic model assessment of insulin resistance.

### 3.7 Risk of bias and quality of methods

**FIGURE 5:**
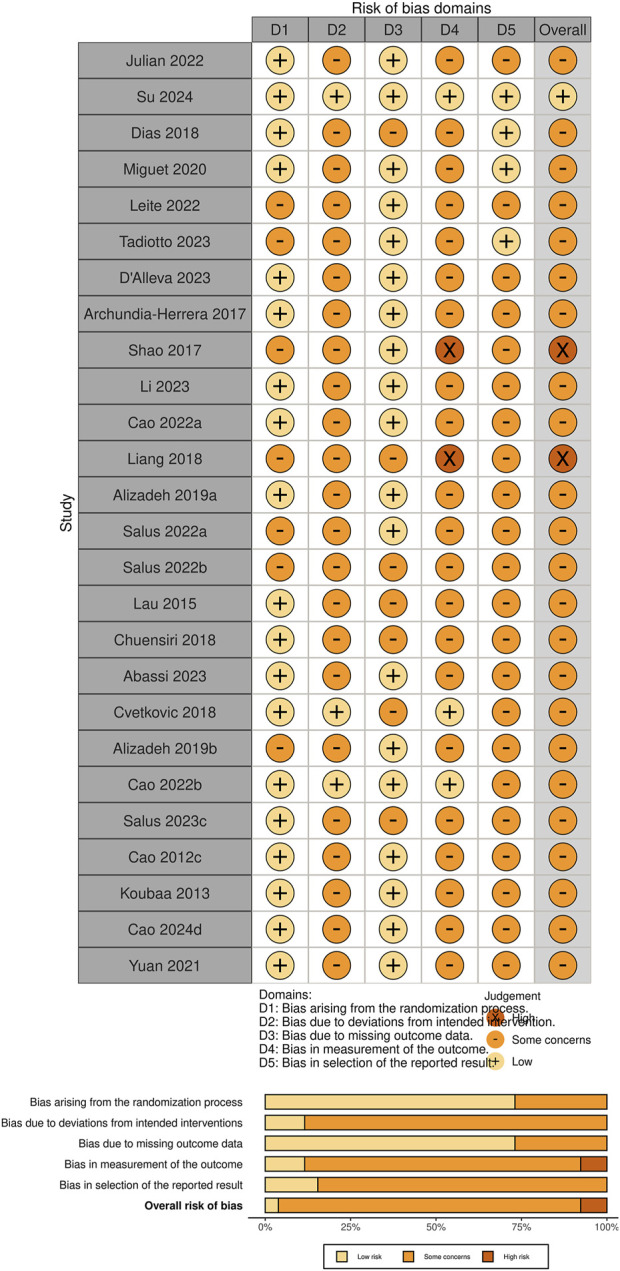
Risk of bias for the included studies.

Figure 5 illustrates the risk of bias assessment across different domains for the included studies. Approximately 75% of the studies were rated as low risk of bias in the domains of “randomization process” and “missing outcome data,” indicating adequate control during the initial study design and outcome completeness. However, a considerable number of studies showed “some concerns” in the domains of “deviations from intended interventions” and “measurement of the outcome,” suggesting that the risk of detection bias should not be overlooked. Overall, most studies were judged to have “some concerns” regarding risk of bias, while only a few studies (e.g., Shao and Fei, 2017; Liang and Hao, 2018) were rated as high risk, primarily due to the lack of blinding of outcome assessors, which may compromise the validity of subjectively measured outcomes.

It is worth noting that some studies identified as having a higher risk of bias in the RoB 2 assessment (e.g., Shao and Fei, 2017) still received moderate scores on the PEDro scale. This is mainly attributed to their satisfactory performance in areas such as data completeness, between-group comparisons, and reporting of effect estimates.

The RoB 2 and PEDro tools focus on different methodological dimensions: the former emphasizes bias control and internal validity, while the latter places greater emphasis on the structural quality of trial reporting. Therefore, discrepancies in the scoring outcomes between the two tools are not uncommon. In the present study, we integrated the results from both assessments to provide a more comprehensive evaluation of the methodological quality and reliability of the included studies. Further elaboration is provided in the Discussion section.

Publication bias was assessed using funnel plots in combination with Egger’s test to examine the potential bias risk in the included studies regarding health outcomes. As the number of included studies was insufficient for most outcomes (n < 10) (Deeks et al., 2005), Egger’s regression was ultimately performed only for the effect of HIIT *versus* MICT on BMI in children and adolescents with overweight or obesity, and no evidence of publication bias was found (p = 0.68). Furthermore, in the original meta-analysis of Body Mass Index (BMI) (11 studies, N = 392), the pooled effect size was (SMD = −0.05, 95% CI [−0.30, 0.20], p = 0.70). The prediction interval was wide (−0.70, 0.60), indicating potential variations across different populations. A trim and fill analysis were conducted to assess publication bias. The adjusted effect size was (SMD = −0.01, 95% CI [-0.27, 0.25], p = 0.94), Indicating a further attenuation of the effect and suggesting that any potential publication bias may have further diminished an already small and insignificant effect. All funnel plots are presented in Supplementary Figures 4–7.

To comprehensively evaluate the certainty of evidence for each primary outcome, we employed the GRADE approach to rate the overall quality of evidence (see Table X). Most outcomes—such as BMI, fat mass, VO_2_max, and blood lipid profiles—were rated as having moderate certainty, indicating a reasonable level of confidence in the estimated effects. However, some outcomes, including waist circumference, insulin, and HOMA-IR, were downgraded to low certainty due to concerns regarding inconsistency among studies or imprecision caused by small sample sizes and wide confidence intervals. Moreover, five outcomes in the comparison between HIIT and no training—including VO_2_max and HDL—were rated as having very low certainty, primarily due to the presence of both serious inconsistency and serious imprecision. These GRADE ratings help contextualize the strength of the evidence and highlight the need for further high-quality research in this area (Table 10).

**TABLE 10:** GRADE criteria for certainty of evidence on (1) the effectiveness of HIIT vs. MICT on health outcomes (2) the effectiveness of HIIT vs. CON and (3) the effectiveness of HIIT vs. CON.

Outcome	Sample size	Certainty of evidence assessment					Hedge’s g † (95% CI)	GRADE*
		Risk of bias	Inconsistency	Indirectness	Imprecision	Others		
HIIT vs. MICT								
BMI	392 (K = 11)	Not serious	Not serious	Not serious	Not serious	None	−0.05 (−0.30, 0.20)	⨁⨁⨁◯ Moderate
Fat mass	265 (K = 6)	Not serious	Not serious	Not serious	Not serious	None	0.13 (−0.27, 0.52)	⨁⨁⨁◯ Moderate
Free fat mass	221 (K = 8)	Not serious	Not serious	Not serious	Not serious	None	0.17 (-0.14, 0.48)	⨁⨁⨁◯ Moderate
Waistline	219 (K = 7)	Not serious	Serious	Not serious	Serious	Low prediction	−0.31 (-0.89, 0.26)	⨁⨁◯◯ Low
Weight	331 (K = 9)	Not serious	Not serious	Not serious	Not serious	None	−0.03 (-0.25, 0.18)	⨁⨁⨁◯ Moderate
Cholesterol	244 (K = 7)	Not serious	Not serious	Not serious	Not serious	None	−0.10 (-0.42, 0.23)	⨁⨁⨁◯ Moderate
Glucose	215 (K = 6)	Not serious	Not serious	Not serious	Not serious	None	0.05 (-0.22, 0.32)	⨁⨁⨁◯ Moderate
Insulin	171 (K = 5)	Not serious	Not serious	Not serious	Serious	Low sample size	0.11 (-0.19, 0.41)	⨁⨁◯◯ Low
Triglyceride	244 (K = 7)	Not serious	Serious	Not serious	Not serious	None	0.00 (-0.44, 0.45)	⨁⨁⨁◯ Moderate
VO2max	249 (K = 7)	Not serious	Not serious	Not serious	Not serious	None	0.81 (0.33, 1.28)	⨁⨁⨁◯ Moderate
DBP	179 (K = 6)	Not serious	Not serious	Not serious	Not serious	None	−0.22 (-0.51, 0.08)	⨁⨁⨁◯ Moderate
SBP	179 (K = 6)	Not serious	Not serious	Not serious	Not serious	None	−0.51 (-0.87, −0.15)	⨁⨁⨁◯ Moderate
HDL	244 (K = 7)	Not serious	Serious	Not serious	Not serious	None	−0.07 (-0.80, 0.65)	⨁⨁⨁◯ Moderate
LDL	204 (K = 6)	Not serious	Not serious	Not serious	Not serious	None	−0.14 (-0.67, 0.38)	⨁⨁⨁◯ Moderate
HOMA-IR	175 (K = 5)	Not serious	Not serious	Not serious	Serious	Low sample size	0.02 (-0.28, 0.32)	⨁⨁◯◯ Low
HIIT vs. No Training								
BMI	230 (K = 8)	Not serious	Serious	Not serious	Not serious	None	−1.75 (-3.65, 0.15)	⨁⨁⨁◯ Moderate
Waistline	115 (K = 4)	Not serious	Not serious	Not serious	Serious	Low sample size	−0.67 (-1.05, −0.29)	⨁⨁◯◯ Low
Weight	256 (K = 9)	Not serious	Not serious	Not serious	Not serious	None	−0.81 (-1.25, −0.36)	⨁⨁⨁◯ Moderate
Fat mass	93 (K = 3)	Not serious	Not serious	Serious	Serious	Low sample size	−0.69 (-1.37, −0.01)	⨁⨁◯◯ Low
VO_2_max	158 (K = 5)	Not serious	Serious	Not serious	Serious	Low sample size	2.06 (1.00, 3.12)	⨁◯◯◯ very Low
DBP	75 (K = 3)	Not serious	Not serious	Serious	Serious	Low sample size	−0.68 (-1.15, −0.21)	⨁⨁◯◯ Low
SBP	75 (K = 3)	Not serious	Serious	Not serious	Serious	Low sample size	−0.17 (-1.25, 0.92)	⨁◯◯◯ very Low
HDL	90 (K = 3)	Not serious	Serious	Not serious	Serious	Low sample size	0.00 (-0.65, 0.64)	⨁◯◯◯ very Low
LDL	90 (K = 3)	Not serious	Serious	Not serious	Serious	Low sample size	−0.58 (-1.24, 0.08)	⨁◯◯◯ very Low
Cholesterol	90 (K = 3)	Not serious	Serious	Not serious	Serious	Low sample size	−0.44 (-1.31, 0.43)	⨁◯◯◯ very Low
MICT vs. No Training								
BMI	124 (K = 6)	Not serious	Not serious	Not serious	Not serious	None	−1.58 (-2.96, −0.20)	⨁⨁⨁◯ Moderate
Fat mass	107 (K = 6)	Not serious	Not serious	Not serious	Not serious	None	−1.24 (-2.48, 0.01)	⨁⨁⨁◯ Moderate
Free fat mass	76 (K = 4)	Not serious	Not serious	Not serious	Not serious	None	−0.15 (-0.76,0.46)	⨁⨁⨁◯ Moderate
Waistline	66 (K = 4)	Not serious	Serious	Not serious	Serious	Low prediction	−0.43 (-1.09,0.22)	⨁⨁◯◯ Low
Weight	99 (K = 5)	Not serious	Not serious	Not serious	Not serious	None	−0.59 (-1.00, −0.18)	⨁⨁⨁◯ Moderate
Cholesterol	73 (K = 4)	Not serious	Not serious	Not serious	Not serious	None	−0.30 (-0.86,0.27)	⨁⨁⨁◯ Moderate
Glucose	73 (K = 4)	Not serious	Not serious	Not serious	Not serious	None	−0.21 (-0.96,0.54)	⨁⨁⨁◯ Moderate
Insulin	51 (K = 3)	Not serious	Not serious	Not serious	Serious	Low sample size	−0.81 (-2.10,0.49)	⨁⨁◯◯ Low
Triglyceride	73 (K = 4)	Not serious	Serious	Not serious	Not serious	None	−0.25 (-1.17,0.66)	⨁⨁⨁◯ Moderate
VO_2_max	108 (K = 6)	Not serious	Not serious	Not serious	Not serious	None	1.26 (0.61,1.90)	⨁⨁⨁◯ Moderate
DBP	71 (K = 4)	Not serious	Not serious	Not serious	Not serious	None	−0.60 (-1.10, −0.10)	⨁⨁⨁◯ Moderate
SBP	71 (K = 4)	Not serious	Not serious	Not serious	Not serious	None	−0.80 (-1.99,0.38)	⨁⨁⨁◯ Moderate
HDL	73 (K = 4)	Not serious	Serious	Not serious	Not serious	None	−0.16 (-1.09,0.76)	⨁⨁⨁◯ Moderate
LDL	53 (K = 3)	Not serious	Not serious	Not serious	Not serious	None	−0.21 (-1.45,1.02)	⨁⨁⨁◯ Moderate
HOMA-IR	73 (K = 4)	Not serious	Not serious	Not serious	Serious	Low sample size	−0.54 (-1.66,0.57)	⨁⨁◯◯ Low

K, the total number of effects included in the pooled effect size; VO2max, Maximal Oxygen Uptake; DBP, diastolic blood pressure; SBP, systolic blood pressure; HDL, High-Density Lipoprotein; LDL, Low-Density Lipoprotein; HOMA-IR, homeostatic model assessment of insulin resistance.

*GRADE, Criteria for Certainty of Evidence.

High, Very confident in the estimated effect.

Moderate, Moderately confident in the estimated effect.

Low, Limited confidence in the estimated effect.

Very low, Very limited confidence in the estimated effect.

### 3.8 Sensitivity analysis

To assess the robustness of the findings in this study, we applied a two-level random-effects model and the leave-one-out method to examine the influence of individual studies on the overall effect estimates. During the sensitivity analysis, each study was removed one at a time, and the overall effect size was recalculated to observe any changes in the results.

We further examined the influence of studies identified as having a high risk of bias or small sample sizes, based on PEDro scores, RoB 2 assessments, and predefined sample size thresholds. Specifically, studies with PEDro scores ≤4, RoB 2 ratings of “high,” or fewer than 10 participants per group were included (e.g., Shao and Fei, 2017; Liang and Hao, 2018; Lau et al., 2015; Salus et al., 2022a). In the leave-one-out sensitivity analysis (Supplementary Figures S8–S10), the exclusion of most of these studies did not materially alter the pooled effect sizes or confidence intervals. However, we observed that in the comparison between HIIT and no training, omitting (Salus et al., 2022b) resulted in statistically significant pooled effects for several outcomes, including cholesterol, DBP, SBP, fat mass, and LDL. This suggests that the study had a considerable influence on these outcomes. Nevertheless, the direction of the pooled effects remained consistent, and therefore, the overall conclusions of the meta-analysis can still be considered robust.

## 4 Discussion

This study focuses on children and adolescents with overweight or obesity, with a more comprehensive evaluation of health-related outcomes, providing stronger specificity and reference value. The main findings indicate that both HIIT and MICT significantly improve body composition and cardiorespiratory health indicators in children and adolescents with overweight or obesity, compared with no exercise. Specifically, HIIT interventions led to nole reductions in weight, waist circumference, and fat mass, along with marked improvements in VO_2_max and DBP, suggesting cardiovascular benefits. Similarly, MICT significantly improved BMI, weight, fat mass, VO_2_max, and DBP, with robust findings across outcomes. It appeared particularly effective in reducing weight and fat mass and enhancing cardiorespiratory endurance. When directly comparing HIIT and MICT, HIIT showed superior effects on cardiopulmonary fitness and cardiovascular risk factors. In particular, HIIT yielded greater improvements in VO_2_max and a significant reduction in SBP, both confirmed by sensitivity analyses. Additionally, factors such as weight status, sex, age, training frequency, and training modality may moderate the effectiveness of HIIT interventions.

### 4.1 Effects of HIIT on health outcomes

Our meta-analysis showed that, compared with a non-exercise control group, HIIT had marked effects on weight, waist circumference, fat mass, DBP, and VO_2_max. Our findings for VO_2_max and DBP are consistent with previous studies (Costigan et al., 2015; Martin-Smith et al., 2020; Men et al., 2023; Deng and Wang, 2024), thereby reinforcing the positive impact of HIIT on cardiopulmonary health. However, our results also showed consistent improvements in weight, waist circumference, and fat mass following HIIT in children and adolescents with overweight or obesity, while previous meta-analyses have yielded inconsistent findings regarding these body composition outcomes. For example, Costigan et al. (2015)reported effects of HIIT on fat mass consistent with our findings, but their conclusion on waist circumference differed, possibly due to variations in study populations. In the meta-analysis by Men et al. (2023), which examined the effects of HIIT *versus* no training on body composition and cardiometabolic outcomes in children and adolescents, the results were in direct contrast to ours. A closer review of their included studies suggests that the inclusion of trials comparing HIIT with MICT may have influenced their overall findings. In contrast, our study more clearly distinguished between these comparisons. The heterogeneity for body composition indicators in our analyses was moderate, and sensitivity analyses confirmed the robustness of our findings. Currently, consensus statements (Ross et al., 2020) recognize waist circumference as a key marker and primary driver of cardiometabolic risk regardless of BMI, age, or sex, and recommend its clinical application. In our study, the pooled effect size for waist circumference was SMD = −0.67, which falls within the moderate-to-large range based on Cohen’s criteria, suggesting that HIIT may exert a clinically meaningful effect on abdominal adiposity in children and adolescents with overweight or obesity. As for the physiological mechanisms underlying the effects of HIIT on body composition, they may be related to mitochondrial adaptations (Henríquez-Olguín et al., 2019) and the excess post-exercise oxygen consumption (EPOC) effect (Valéria et al., 2021).

Our subgroup analysis explored potential factors influencing the relationship between HIIT and health outcomes, focusing on overweight or obesity status, age differences, sex, intervention modality, and intervention duration. Due to the limited number of included studies, the results only reflect the effects of HIIT on body composition and maximal oxygen uptake (VO_2_max). The findings showed that overweight or obesity status had a statistically significant impact on improvements in VO_2_max (p < 0.05). However, it is important to note that this conclusion is based on only one study involving participants with obesity, and thus requires further confirmation through additional research. No other subgroup differences reached statistical significance (p_b_>0.05). Nevertheless, the overall results suggest that HIIT may yield positive health benefits regardless of weight status, age, sex, or training modality. In addition, based on the distribution of studies included in the subgroup analyses, it is evident that most research has focused on body composition, cardiorespiratory fitness, overweight status, and male participants, while placing less emphasis on more diverse health indicators, more refined BMI classifications, and female populations.

### 4.2 Effects of MICT on health outcomes

Our findings showed that, compared with a non-exercise control, MICT significantly improved weight, BMI, VO_2_max, and DBP. These results are consistent with previous studies, supporting the effectiveness of MICT in regulating body composition and enhancing cardiorespiratory fitness (Thorogood et al., 2011; Kelley et al., 2019; O’Donoghue et al., 2021). It should be noted that the populations included in this meta-analysis were children and adolescents with overweight or obesity; therefore, the observed effect sizes may not be generalizable to individuals with normal weight.

To further explore potential moderators influencing the outcomes, we conducted subgroup analyses based on different BMI classifications, age, sex, and training mode. The results showed that only weight was significantly influenced by overweight or obesity status, while no other factors demonstrated a moderating effect. This may be attributed to the fact that individuals with obesity typically have a higher basal metabolic load and greater fat accumulation compared to those who are overweight (Griffiths et al., 1990). However, the underlying mechanisms behind this difference are beyond the scope of the present review.

### 4.3 Effects of HIIT and MICT on health outcomes

Our study found only minor differences between HIIT and MICT, primarily in SBP and VO_2_max. This finding is consistent with previous research, which has also reported differential effects between HIIT and MICT on SBP (Thivel et al., 2019; Way et al., 2019) and VO_2_max (García-Hermoso et al., 2016; Thivel et al., 2019).

With regard to VO_2_max, our findings are consistent with previous studies (Cao et al., 2019; Deng and Wang, 2024; Poon et al., 2024b; Wang et al., 2024), indicating that HIIT provides greater improvements than both no exercise and MICT. This may be attributed to the previously mentioned mechanisms such as mitochondrial adaptations (Henríquez-Olguín et al., 2019), as well as improvements in hemoglobin levels that enhance oxygen transport capacity (Helgerud et al., 2007). By contrast, there remains some controversy regarding SBP. Several studies (Ramos et al., 2015; Liang et al., 2024) have reported no significant difference between HIIT and MICT in their effects on SBP. This discrepancy may be related to differences in study populations and intervention protocols. For example, in the study by Liang et al. (2024), subgroup analysis revealed that sprint interval training (SIT) was more effective in reducing SBP when the intervention duration was ≥8 weeks or the sprint time was <30 s. Similarly, Short et al. (2012) suggested that HIIT may lower blood pressure more effectively than MICT, possibly due to greater post-exercise oxygen consumption (EPOC) and increased nitric oxide production, which helps reduce vascular resistance.

To investigate the sources of variation, we conducted subgroup analyses. The results indicated that differences in SBP outcomes among children and adolescents with overweight or obesity were associated with weight status and sex. Although no statistically significant differences were found between HIIT and MICT across subgroups defined by age, frequency, or intervention modality, the results showed that HIIT was more effective in reducing SBP among adolescents than among children (p < 0.01). This finding is supported by a Canadian adolescent cohort study, which reported similar results (Maximova et al., 2009). Therefore, it is possible that the observed effects of HIIT on SBP are particularly pronounced in male adolescents with obesity. Our results also showed that while there was no statistically significant difference in the effect of running compared to cycling on SBP, both modalities approached statistical significance. Cycling (p = 0.05) and running (p = 0.09) both appeared to be effective strategies for blood pressure regulation. Given that the participants in our study were children and adolescents with overweight or obesity, and considering the potential risk of knee joint stress associated with running (Colberg et al., 2010; Rynecki et al., 2019), we recommend cycling as a more appropriate intervention for this population. In addition, the findings suggest that a training frequency of three sessions per week is effective in reducing SBP. These results provide useful insights for designing exercise prescriptions aimed at preventing hypertension in children and adolescents with overweight or obesity.

Our findings revealed that sex and age significantly moderated HDL levels, while sex also influenced LDL levels. Although no significant differences were found between HIIT and MICT for lipoprotein outcomes, this is consistent with previous studies (McCORMICK et al., 2023; Chen et al., 2025). However, subgroup analyses in our study suggested that sex and age may influence HDL and LDL levels, even though the related findings in earlier research were based on adult populations. Existing theories indicate that exercise can regulate sex hormones differently between males and females (Monninkhof et al., 2007; Hawkins et al., 2008). For instance, estrogen has been shown to promote HDL synthesis (Imamoglu and Yildirim, 2005; Palmisano et al., 2018), thereby preventing atherosclerosis and improving cardiovascular health. In our study, HDL decreased and LDL increased among male participants. Although these changes may seem unfavorable, they could indicate enhanced lipid metabolism following exercise (Goldberg and Elliot, 1987). The LDL increase may result from elevated lipoprotein lipase activity, which enhances VLDL metabolism and raises circulating LDL levels (Chen et al., 2025). In other words, reductions in HDL and increases in LDL after exercise are not necessarily negative outcomes, as they may indicate underlying improvements in lipid turnover and overall metabolic function. In mixed-sex groups, trends may be influenced by female participants’ metabolic responses. Unfortunately, due to the limited number of studies focusing on female participants, further conclusions could not be drawn. These observations suggest that sex may indeed influence the effect of exercise on lipoproteins in children and adolescents with overweight or obesity, although further original research is needed to confirm this. In addition, Our findings on age-related effects align with Eisenmann, (2002), even though their sample consisted of adolescent athletes. The impact of pubertal hormone changes on children and adolescents remains relevant. The reduction in HDL observed during adolescence may be related to increases in testosterone and the redistribution of body fat (Baumgartner et al., 1989; Boisseau and Delamarche, 2000). Although our findings did not reach statistical significance for HDL among adolescents, the direction of the effect (SMD = −0.42) suggests a downward trend. Similarly, the age-related influence on LDL levels is also likely driven by hormonal changes (Eisenmann, 2002). In summary, sex and age are likely key moderators of lipoprotein changes in children and adolescents with overweight or obesity. Further research should refine age classifications and explore sex-specific responses to exercise.

### 4.4 Bias and quality implications

While the Results section provided a descriptive account of risk of bias and methodological quality assessments, further interpretation is warranted regarding how these quality issues may have impacted the pooled results and the certainty of evidence. Most of the included studies were judged as having “some concerns” in the RoB 2 evaluation, particularly in domains related to deviations from intended interventions and outcome measurement. These domains are especially relevant in exercise intervention trials, where blinding is often impractical. The presence of detection bias may affect the objectivity of outcome assessments, particularly for self-reported or subjectively measured variables such as perceived exertion or adherence.

Although the majority of studies were rated as moderate quality on the PEDro scale, the limitations identified through RoB 2 were explicitly considered when grading the overall certainty of evidence using the GRADE framework. For example, outcomes such as VO_2_max, DBP, and fat mass in the HIIT *versus* no training comparison were downgraded to “very low” certainty due to a combination of high risk of bias and imprecision arising from small sample sizes or wide confidence intervals. These downgrades reflect a cautious interpretation of findings, even in cases where the effect sizes were statistically significant.

Moreover, we recognize that studies with high or unclear risk of bias may contribute disproportionately to heterogeneity in the meta-analysis. While sensitivity analyses confirmed the robustness of our main findings, the potential influence of lower-quality studies cannot be ruled out. Therefore, we strongly recommend that future RCTs in this field improve methodological rigor, particularly in blinding procedures and standardization of outcome assessment protocols, to strengthen the quality and reliability of evidence in exercise interventions among children and adolescents with overweight or obesity.

### 4.5 Practical applications and limitations

This study highlights that both HIIT and MICT are effective in improving health outcomes, with only minor differences observed between the two. If the focus is on cardiopulmonary benefits and time efficiency, HIIT may be preferable. However, exercise programs should be tailored to the needs of specific populations. Some studies suggest that the practical implementation of HIIT in schools can be achieved through enjoyable formats tailored to students’ fitness levels, and that HIIT programs can be conducted without disrupting regular school schedules, thereby supporting their feasibility (Delgado-Floody et al., 2019; Pérez-Ramírez et al., 2025). Regarding safety, current evidence indicates that HIIT is generally safe for children and adolescents (Liu et al., 2024); however, the lack of standardized injury reporting mechanisms in most studies warrants cautious interpretation. In addition, although some of the included studies had intervention durations as short as 3 weeks and the subgroup analysis showed no significant differences based on training duration, broader evidence from the literature suggests that a duration of around 8 weeks may be more appropriate. Therefore, the practical application of training duration remains uncertain. Our recommendation is as follows.

First, a school-based HIIT program could be implemented three times per week, with an intensity ranging from 85% of HRpeak to maximal effort, interval durations of 20 s to 4 min, and recovery periods of 60–240 s. In addition, continuous exercise lasting more than 40 min is recommended during after-school hours, preferably in the form of outdoor cycling, which is more joint-friendly for individuals with overweight or obesity. The frequency of these Exercise programs can be adjusted based on academic demands, with at least two sessions per week suggested. It should be noted that this proposed plan is not a one-size-fits-all solution; more personalized interventions should be developed according to individual conditions and practical circumstances. In addition, some practical strategies are might recommended to further enhance the feasibility and effectiveness of HIIT in youth populations. Combining HIIT with other physiological stimuli such as blood flow restriction may allow individuals with obesity to experience similar cardiometabolic benefits at lower intensities (Yin et al., 2025). Second, distributing short HIIT bouts throughout the day as accumulated exercise has been demonstrated to be an effective and time-efficient strategy in school settings, helping to integrate physical activity seamlessly into students’ daily routines (Yin et al., 2024d; 2024c; 2024b).

Nevertheless, several limitations should be acknowledged. Although subgroup and sensitivity analyses were conducted, the limited number of included studies and the relatively low proportion of high-quality research may have introduced potential publication bias. In particular, the lack of studies focusing on health outcomes in females restricted our ability to fully explore the effects of HIIT and MICT in children and adolescents with overweight or obesity. Additionally, variations in testing methods across studies may have introduced measurement bias that could have affected the results.

## 5 Conclusion

This meta-analysis confirmed that both HIIT and MICT improve body composition and cardiopulmonary health in children and adolescents with overweight or obesity. HIIT was more effective than MICT in improving VO_2_max and reducing SBP, particularly in subgroups such as overweight children, obese male adolescents, and programs with more than three sessions per week. Although running-based HIIT demonstrated the strongest effect on VO_2_max, cycling is recommended as a safer alternative in real-world settings. Given the limited number of studies in some subgroups and the potential for bias, these results should be interpreted with caution. Nonetheless, the findings provide valuable insights for tailoring exercise prescriptions to specific populations and contexts.
