# Supplementary material for: Effects and moderator of high-intensity interval training and moderate-intensity continuous training among children and adolescents with overweight or obese: a systematic review and meta-analysis
Source: Front Physiol. 2025 Jul 30;16:1625516. doi: 10.3389/fphys.2025.1625516 (PMC12343602; doi:10.3389/fphys.2025.1625516)
Supplement: Supplementary file 2 [file Supplementaryfile1.docx]

**Electronic Supplementary Material**

**Electronic Supplementary Material PRISMA checklist**

| **Section and Topic** | **Item #** | **Checklist item** | **Location where item is reported** |
| --- | --- | --- | --- |
| **TITLE** | | |  |
| Title | 1 | Identify the report as a systematic review. | Title |
| **ABSTRACT** | | |  |
| Abstract | 2 | See the PRISMA 2020 for Abstracts checklist. | Abstract |
| **INTRODUCTION** | | |  |
| Rationale | 3 | Describe the rationale for the review in the context of existing knowledge. | 1.Introduction |
| Objectives | 4 | Provide an explicit statement of the objective(s) or question(s) the review addresses. |  |
| **METHODS** | | |  |
| Eligibility criteria | 5 | Specify the inclusion and exclusion criteria for the review and how studies were grouped for the syntheses. | 2.1-2.7 |
| Information sources | 6 | Specify all databases, registers, websites, organisations, reference lists and other sources searched or consulted to identify studies. Specify the date when each source was last searched or consulted. |  |
| Search strategy | 7 | Present the full search strategies for all databases, registers and websites, including any filters and limits used. |  |
| Selection process | 8 | Specify the methods used to decide whether a study met the inclusion criteria of the review, including how many reviewers screened each record and each report retrieved, whether they worked independently, and if applicable, details of automation tools used in the process. |  |
| Data collection process | 9 | Specify the methods used to collect data from reports, including how many reviewers collected data from each report, whether they worked independently, any processes for obtaining or confirming data from study investigators, and if applicable, details of automation tools used in the process. |  |
| Data items | 10a | List and define all outcomes for which data were sought. Specify whether all results that were compatible with each outcome domain in each study were sought (e.g. for all measures, time points, analyses), and if not, the methods used to decide which results to collect. |  |
|  | 10b | List and define all other variables for which data were sought (e.g. participant and intervention characteristics, funding sources). Describe any assumptions made about any missing or unclear information. |  |
| Study risk of bias assessment | 11 | Specify the methods used to assess risk of bias in the included studies, including details of the tool(s) used, how many reviewers assessed each study and whether they worked independently, and if applicable, details of automation tools used in the process. |  |
| Effect measures | 12 | Specify for each outcome the effect measure(s) (e.g. risk ratio, mean difference) used in the synthesis or presentation of results. |  |
| Synthesis methods | 13a | Describe the processes used to decide which studies were eligible for each synthesis (e.g. tabulating the study intervention characteristics and comparing against the planned groups for each synthesis (item #5)). |  |
|  | 13b | Describe any methods required to prepare the data for presentation or synthesis, such as handling of missing summary statistics, or data conversions. |  |
|  | 13c | Describe any methods used to tabulate or visually display results of individual studies and syntheses. |  |
|  | 13d | Describe any methods used to synthesize results and provide a rationale for the choice(s). If meta-analysis was performed, describe the model(s), method(s) to identify the presence and extent of statistical heterogeneity, and software package(s) used. |  |
|  | 13e | Describe any methods used to explore possible causes of heterogeneity among study results (e.g. subgroup analysis, meta-regression). |  |
|  | 13f | Describe any sensitivity analyses conducted to assess robustness of the synthesized results. |  |
| Reporting bias assessment | 14 | Describe any methods used to assess risk of bias due to missing results in a synthesis (arising from reporting biases). |  |
| Certainty assessment | 15 | Describe any methods used to assess certainty (or confidence) in the body of evidence for an outcome. |  |
| **RESULTS** | | |  |
| Study selection | 16a | Describe the results of the search and selection process, from the number of records identified in the search to the number of studies included in the review, ideally using a flow diagram. | 3.1-3.8 |
|  | 16b | Cite studies that might appear to meet the inclusion criteria, but which were excluded, and explain why they were excluded. |  |
| Study characteristics | 17 | Cite each included study and present its characteristics. |  |
| Risk of bias in studies | 18 | Present assessments of risk of bias for each included study. |  |
| Results of individual studies | 19 | For all outcomes, present, for each study: (a) summary statistics for each group (where appropriate) and (b) an effect estimate and its precision (e.g. confidence/credible interval), ideally using structured tables or plots. |  |
| Results of syntheses | 20a | For each synthesis, briefly summarise the characteristics and risk of bias among contributing studies. |  |
|  | 20b | Present results of all statistical syntheses conducted. If meta-analysis was done, present for each the summary estimate and its precision (e.g. confidence/credible interval) and measures of statistical heterogeneity. If comparing groups, describe the direction of the effect. |  |
|  | 20c | Present results of all investigations of possible causes of heterogeneity among study results. |  |
|  | 20d | Present results of all sensitivity analyses conducted to assess the robustness of the synthesized results. |  |
| Reporting biases | 21 | Present assessments of risk of bias due to missing results (arising from reporting biases) for each synthesis assessed. |  |
| Certainty of evidence | 22 | Present assessments of certainty (or confidence) in the body of evidence for each outcome assessed. |  |
| **DISCUSSION** | | |  |
| Discussion | 23a | Provide a general interpretation of the results in the context of other evidence. | 4-4.5 |
|  | 23b | Discuss any limitations of the evidence included in the review. |  |
|  | 23c | Discuss any limitations of the review processes used. |  |
|  | 23d | Discuss implications of the results for practice, policy, and future research. |  |
| **OTHER INFORMATION** | | |  |
| Registration and protocol | 24a | Provide registration information for the review, including register name and registration number, or state that the review was not registered. | Abstract |
|  | 24b | Indicate where the review protocol can be accessed, or state that a protocol was not prepared. | N/a |
|  | 24c | Describe and explain any amendments to information provided at registration or in the protocol. | N/a |
| Support | 25 | Describe sources of financial or non-financial support for the review, and the role of the funders or sponsors in the review. | Funding |
| Competing interests | 26 | Declare any competing interests of review authors. | Declaration of competing interest |
| Availability of data, code and other materials | 27 | Report which of the following are publicly available and where they can be found: template data collection forms; data extracted from included studies; data used for all analyses; analytic code; any other materials used in the review. | N/a |

*From:*  Page MJ, McKenzie JE, Bossuyt PM, Boutron I, Hoffmann TC, Mulrow CD, et al. The PRISMA 2020 statement: an updated guideline for reporting systematic reviews. BMJ 2021;372:n71. doi: 10.1136/bmj.n71. This work is licensed under CC BY 4.0. To view a copy of this license, visit <https://creativecommons.org/licenses/by/4.0/>

**Electronic Supplementary Material Table.1:** Search Strategy (PUBMED and Web of Science)

| 1. **Children, teenagers**   "child" OR "children" OR "adolescent" OR "adolescents" OR "youth" OR "teenager" OR "teenagers" OR "pediatric" OR "paediatric" OR "juvenile" OR "obese children" OR "overweight children" OR "obese adolescents" OR "overweight adolescents" OR "childhood obesity" OR "pediatric obesity" OR "adolescent obesity" OR "youth obesity" OR "overweight youth" OR "childhood overweight" OR "BMI in children" OR "BMI in adolescents" OR "excess weight in children" OR "weight status in youth" OR "obesity in youth" OR "body mass index in children" OR "body mass index in adolescents" |
| --- |
| 1. **High-Intensity Interval Training**   "interval training" OR "high-intensity interval training" OR "high intensity interval training" OR "high-intensity interval trainings" OR "interval training, high-intensity" OR "interval trainings, high-intensity" OR "trainings, high-intensity interval" OR "high-intensity intermittent exercise" OR "exercise, high-intensity intermittent" OR "exercises, high-intensity intermittent" OR "high-intensity intermittent exercises" OR "sprint interval training" OR "sprint interval trainings" OR "aerobic interval training" OR "high-volume interval training" OR "repeated sprint training" or "intermittent exercise training" OR "HIIT" OR "SIT" OR "HIT" OR "HIIE" OR "AIT" OR "RST" OR "IET" |
| 1. **Moderate-intensity continuous training**   "moderate-intensity continuous training" OR "MCT" OR "continuous aerobic training" OR "steady-state exercise" OR "endurance training" OR "moderate-intensity aerobic exercise" OR "continuous training" OR "submaximal exercise" |
| 1. **Cardiometabolic Health and Body Composition**   "left ventricular function" OR "systolic function" OR "diastolic function" OR "blood pressure" OR "systolic blood pressure" OR "diastolic blood pressure" OR "mean arterial pressure" OR "heart rate" OR "heart rate variability" OR "pulse rate" OR "cardiovascular function" OR "cardiovascular" OR "cardiorespiratory fitness" OR "aerobic capacity" OR "metabolism" OR "metabolic health" OR "body composition" OR "physical fitness" OR "health-related fitness" OR "endurance" OR "cardiovascular health" OR "cardiovascular fitness" OR "VO2 max" OR "aerobic fitness" OR "physical health" OR "muscular fitness" OR "glucose" OR "glycaemia" OR "glycemia" OR "glucose intolerance" OR "insulin" OR "insulin sensitivity" OR "insulin resistance" OR "lipid" OR "lipaemia" OR "lipemia" OR "triglycerides" OR "triglyceride" OR "triacylglycerol" OR "triacylglyceride" OR "metabolic" OR "cardiometabolic risk" OR "blood lipid" OR "cholesterol" OR "LDL cholesterol" OR "HDL cholesterol" OR "total cholesterol" OR "blood fat" OR "lipoprotein" OR "apolipoprotein" OR "body composition" OR "lean body mass" OR "fat mass" OR "body fat percentage" OR "body mass index" OR "BMI" OR "waist circumference" OR "waist-to-hip ratio" OR "adiposity" OR "muscle mass" OR "bone density" OR "body weight" OR "anthropometry" OR "body fat distribution" |

**Electronic Supplementary Material Table.2:**Search Strategy (CNKI and CSTJ)

| **儿童青少年**  儿童+青少年+肥胖儿童+超重儿童+肥胖青少年+超重青少年+久坐儿童+久坐青少年 |
| --- |
| **高强度间歇训练**  间歇训练 + 高强度间歇训练 + 低容量高强度间歇训练 + 高强度间歇运动 + 高强度间歇性训练 + 大强度间歇训练 |
| **中等强度持续训练**  持续训练 + 中等强度持续训练 + 持续中等强度有氧训练 +中等强度持续运动 |
| **心脏代谢健康与身体成分**  心脏功能 + 血压 + 收缩压 + 舒张压 + 平均动脉压 + 心率变异性 + 心血管功能 + 心血管 + 心肺适能 + 有氧能力 + 新陈代谢 + 代谢健康 + 肌肉适能 + 心血管健康 + 柔韧性 + 最大摄氧量 + 有氧适能 + 体质健康 + 葡萄糖 + 血糖 + 胰岛素 + 胰岛素敏感性 + 胰岛素抵抗 + 脂质 + 血脂 + 三酰甘油 + 代谢 + 葡萄糖 + 葡萄糖耐量 + 脂质 + 脂质 + 甘油三酯 + 心脏代谢风险 + 胆固醇 + 低密度脂蛋白胆固醇 + 高密度脂蛋白胆固醇 + 脂肪 + 脂蛋白 + 载脂蛋白 + 身体成分 + 瘦体重 + 脂肪量 + 体脂百分比 + 体重 + BMI + 腰围 + 腰臀比 + 肥胖 |

**Electronic Supplementary Material**

**Fig 1.** Summary of the impact of HIIT vs CON on health outcomes

**Electronic Supplementary Material**

**Fig 2.** Summary of the impact of MICT vs CON on health outcomes

**Electronic Supplementary Material**

**Fig 3.**Summary of the impact of HIIT vs MICT on health outcomes

**Electronic Supplementary Material**

**Fig 4.**Funnel plot of the effects of HIIT vs CON on health outcomes

**Electronic Supplementary Material**

**Fig 5.**Funnel plot of the effects of MICT vs CON on health outcomes

**Electronic Supplementary Material**

**Fig 6.**Funnel plot of the effects of HIIT vs MICT on health outcomes

**Fig 7.**Funnel plotof the effects of HIIT vs MICT on Trim-and-Fill (BMI)

**Electronic Supplementary Material**

**Fig 8.**Sensitivity analysis for the effects of HIIT vs CON on health outcomes

**Electronic Supplementary Material**

**Fig 9.**Sensitivity analysis for the effects of MICT vs CON on health outcomes

**Electronic Supplementary Material**

**Fig 10.**Sensitivity analysis for the effects of HIIT vs MICT on health outcomes

**Electronic Supplementary Material Table.3(**Cycle-based subgroup analysis of health outcomes in HIIT vs MICT)

| Outcomes | Subgroup | K | Hedges' *g* | 95% CI | *p*_v_ | *I*^2^ | *p*_b_ |
| --- | --- | --- | --- | --- | --- | --- | --- |
| BMI | ≤ 8 weeks | 1 | -0.14 | [-0.73, 0.45] | n/a | n/a | 0.762 |
|  | > 8 weeks | 10 | -0.04 | [-0.33, 0.25] | 0.78 | 45% |  |
| Weight | ≤ 8 weeks | 1 | -0.09 | [-0.68, 0.50] | n/a | n/a | 0.845 |
|  | > 8 weeks | 8 | -0.02 | [-0.26, 0.21] | 0.84 | 0% |  |
| Cholesterol | ≤ 8 weeks | 1 | 0.49 | [-0.24, 1.22] | n/a | n/a | 0.096 |
|  | > 8 weeks | 6 | -0.19 | [-0.50, 0.13] | 0.25 | 27% |  |
| Glucose | ≤ 8 weeks | 1 | 0.07 | [-0.65, 0.78] | n/a | n/a | 0.969 |
|  | > 8 weeks | 5 | 0.05 | [-0.28, 0.38] | 0.77 | 21% |  |
| Insulin | ≤ 8 weeks | 1 | 0.36 | [-0.37, 1.08] | n/a | n/a | 0.467 |
|  | > 8 weeks | 4 | 0.06 | [-0.27, 0.39] | 0.716 | 0% |  |
| Triglyceride | ≤ 8 weeks | 1 | 0.16 | [-0.56, 0.88] | n/a | n/a | 0.682 |
|  | > 8 weeks | 6 | -0.02 | [-0.54, 0.49] | 0.93 | 72% |  |
| VO2max | ≤ 8 weeks | 1 | 0.27 | [-0.32, 0.86] | n/a | n/a | 0.119 |
|  | > 8 weeks | 6 | 0.91 | [0.37, 1.45] | <0.01 | 70% |  |
| HDL | ≤ 8 weeks | 1 | 0.62 | [-0.12, 1.35] | n/a | n/a | 0.152 |
|  | > 8 weeks | 6 | -0.19 | [-1.01, 0.63] | 0.65 | 88% |  |
| LDL | ≤ 8 weeks | 1 | 0.17 | [-0.54, 0.89] | n/a | n/a | 0.433 |
|  | > 8 weeks | 5 | -0.20 | [-0.81, 0.41] | 0.52 | 74% |  |
| HOMA-IR | ≤ 8 weeks | 1 | 0.37 | [-0.35, 1.09] | n/a | n/a | 0.298 |
|  | > 8 weeks | 4 | -0.05 | [-0.38, 0.28] | 0.76 | 0% |  |

***Note*: *K:*** the total number of effects included in the pooled effect size;***Hedges' g:***the effect size indicators used inthe pooled; ***p*_v_**: overall pooled effect; ***p*_b_**: between subgroups differences; ***95%CI:*** 95% confidence interval; ***I^2^:*** quantitative indicators of heterogeneity;***BMI:***Body Mass Index;***VO2max:***Maximal Oxygen Uptake;***HDL:***High-Density Lipoprotein;***LDL:***Low-Density Lipoprotein;***HOMA-IR:***Homeostatic Model Assessment of Insulin Resistance.

**Electronic Supplementary Material Table.4 Subgroup analysis results of HIIT vs CON**

**Age-based subgroup analysis of health outcomes in HIIT vs No-training**

| Outcomes | Subgroup | K | Hedges' *g* | 95% CI | *p*_v_ | *I*^2^ | *p*_b_ |
| --- | --- | --- | --- | --- | --- | --- | --- |
| BMI | > 12 years | 3 | -2.45 | [-4.67, -0.24] | 0.07 | 93% | 0.263 |
|  | ≤ 12 years | 5 | -1.03 | [-2.18, 0.12] | 0.03 | 93% |  |
| Weight | > 12 years | 4 | -1.03 | [-1.49, -0.56] | ＜0.01 | 24% | 0.393 |
|  | ≤ 12 years | 5 | -0.66 | [-1.36, 0.03] | 0.06 | 74% |  |
| VO2max | > 12 years | 2 | 1.32 | [0.62, 2.01] | ＜0.01 | 40% | 0.121 |
|  | ≤ 12 years | 3 | 2.64 | [1.11, 4.18] | ＜0.01 | 84% |  |

**Cycle-based subgroup analysis of health outcomes in HIIT vs No-training**

| Outcomes | Subgroup | K | Hedges' *g* | 95% CI | *p*_v_ | *I*^2^ | *p*_b_ |
| --- | --- | --- | --- | --- | --- | --- | --- |
| BMI | ≤ 8 weeks | 2 | -4.82 | [-14.33, 4.69] | 0.32 | 96% | 0.431 |
|  | > 8 weeks | 6 | -0.98 | [-2.02, 0.05] | 0.06 | 89% |  |
| Weight | ≤ 8 weeks | 2 | -0.87 | [-2.60, 0.87] | 0.33 | 86% | 0.987 |
|  | > 8 weeks | 7 | -0.85 | [-1.29, -0.41] | ＜0.01 | 56% |  |

**Gender-based subgroup analysis of health outcomes in HIIT vs No-training**

| Outcomes | Subgroup | K | Hedges' *g* | 95% CI | *p*_v_ | *I*^2^ | *p*_b_ |
| --- | --- | --- | --- | --- | --- | --- | --- |
| BMI | Male | 5 | -1.18 | [-2.62, 0.26] | 0.11 | 91% | 0.625 |
|  | Mixed | 3 | -1.75 | [-3.50, 0.01] | 0.05 | 92% |  |
| Weight | Male | 6 | -0.58 | [-1.09, -0.06] | 0.03 | 59% | 0.100 |
|  | Female | 1 | -1.08 | [-1.91, -0.25] | n/a | n/a |  |
|  | Mixed | 2 | -1.43 | [-2.04, -0.83] | ＜0.01 | 17% |  |
| VO2max | Male | 3 | 2.22 | [0.64, 3.80] | ＜0.01 | 88% | 0.597 |
|  | Mixed | 2 | 1.76 | [1.15, 2.38] | ＜0.01 | 4% |  |

**Training Modes subgroup analysis of health outcomes in HIIT vs No-training**

| Outcomes | Subgroup | K | Hedges' *g* | 95% CI | *p*_v_ | *I*^2^ | *p*_b_ |
| --- | --- | --- | --- | --- | --- | --- | --- |
| BMI | Running | 5 | -2.88 | [-6.02, 0.26] | 0.07 | 92% | 0.126 |
|  | Cycling | 3 | -0.15 | [-1.69, 1.40] | 0.853 | 89% |  |
| Weight | Running | 6 | -1.02 | [-1.58, -0.47] | ＜0.01 | 63% | 0.251 |
|  | Cycling | 3 | -0.50 | [-1.20, 0.21] | 0.17 | 62% |  |
| VO2max | Running | 2 | 2.94 | [0.09, 5.79] | 0.04 | 92% | 0.349 |
|  | Cycling | 3 | 1.54 | [0.86, 2.22] | ＜0.01 | 47% |  |

**Electronic Supplementary Material Table.5 Subgroup analysis results of MICT vs CON**

**Age-based subgroup analysis of health outcomes in MICT vs No-training**

| Outcomes | Subgroup | K | Hedges' *g* | 95% CI | *p*_v_ | *I*^2^ | *p*_b_ |
| --- | --- | --- | --- | --- | --- | --- | --- |
| BMI | > 12 years | 2 | -0.68 | [-1.13,-0.02] | ＜0.01 | 0% | 0.136 |
|  | ≤ 12 years | 4 | -2.06 | [-3.82,-0.30] | 0.02 | 95% |  |
| Weight | > 12 years | 2 | -0.75 | [-1.24, -0.26] | 0.11 | 0% | 0.599 |
|  | ≤ 12 years | 3 | -0.52 | [-1.20, 0.15] | 0.13 | 68% |  |
| VO2max | > 12 years | 2 | 1.34 | [0.13, 2.56] | 0.03 | 82% | 0.876 |
|  | ≤ 12 years | 4 | 1.22 | [0.35, 2.10] | ＜0.01 | 83% |  |
| Fat mass | > 12 years | 2 | -0.27 | [-1.30, 0.76] | 0.61 | 76% | 0.135 |
|  | ≤ 12 years | 4 | -1.73 | [-3.35, -0.11] | 0.04 | 93% |  |

**Gender-based subgroup analysis of health outcomes in MICT vs No-training**

| Outcomes | Subgroup | K | Hedges' *g* | 95% CI | *p*_v_ | *I*^2^ | *p*_b_ |
| --- | --- | --- | --- | --- | --- | --- | --- |
| BMI | Male | 4 | -0.61 | [-1.08, -0.15] | ＜0.01 | 0% | 0.107 |
|  | Mixed | 2 | -2.09 | [-3.82, -0.35] | 0.02 | 95% |  |
| Weight | Male | 1 | -0.17 | [-0.83, 0.49] | n/a | n/a | 0.195 |
|  | Mixed | 4 | -0.70 | [-1.17, -0.24] | ＜0.01 | 47% |  |
| VO2max | Male | 2 | 1.42 | [0.35, 2.49] | ＜0.01 | 76% | 0.740 |
|  | Mixed | 4 | 1.19 | [0.31, 2.07] | ＜0.01 | 83% |  |
| Fat mass | Male | 1 | -0.70 | [-1.83,-0.02] | 0.05 | 91% | 0.406 |
|  | Mixed | 5 | -1.34 | [-2.70,0.01] | 0.05 | 93% |  |

**Training Modes subgroup analysis of health outcomes in MICT vs No-training**

| Outcomes | Subgroup | K | Hedges' *g* | 95% CI | *p*_v_ | *I*^2^ | *p*_b_ |
| --- | --- | --- | --- | --- | --- | --- | --- |
| BMI | Running | 4 | -2.08 | [-3.80, -0.36] | 0.02 | 95% | 0.113 |
|  | Cycling | 2 | -0.63 | [-1.10, -0.16] | ＜0.01 | 0% |  |
| Weight | Running | 3 | -0.73 | [-1.37, -0.10] | 0.02 | 65% | 0.394 |
|  | Cycling | 2 | -0.38 | [-0.88, 0.12] | 0.13 | 0% |  |
| VO2max | Running | 4 | 1.51 | [0.50, 2.52] | ＜0.01 | 86% | 0.221 |
|  | Cycling | 2 | 0.81 | [0.33, 1.29] | ＜0.01 | 0% |  |
| Fat mass | Running | 3 | -2.11 | [-4.61,0.38] | 0.10 | 95% | 0.195 |
|  | Cycling | 3 | -0.41 | [-1.08,0.26] | 0.23 | 64% |  |
