# Supplementary figures and images for: Effects and moderator of high-intensity interval training and moderate-intensity continuous training among children and adolescents with overweight or obese: a systematic review and meta-analysis
Source: Front Physiol. 2025 Jul 30;16:1625516. doi: 10.3389/fphys.2025.1625516 (PMC12343602; doi:10.3389/fphys.2025.1625516)

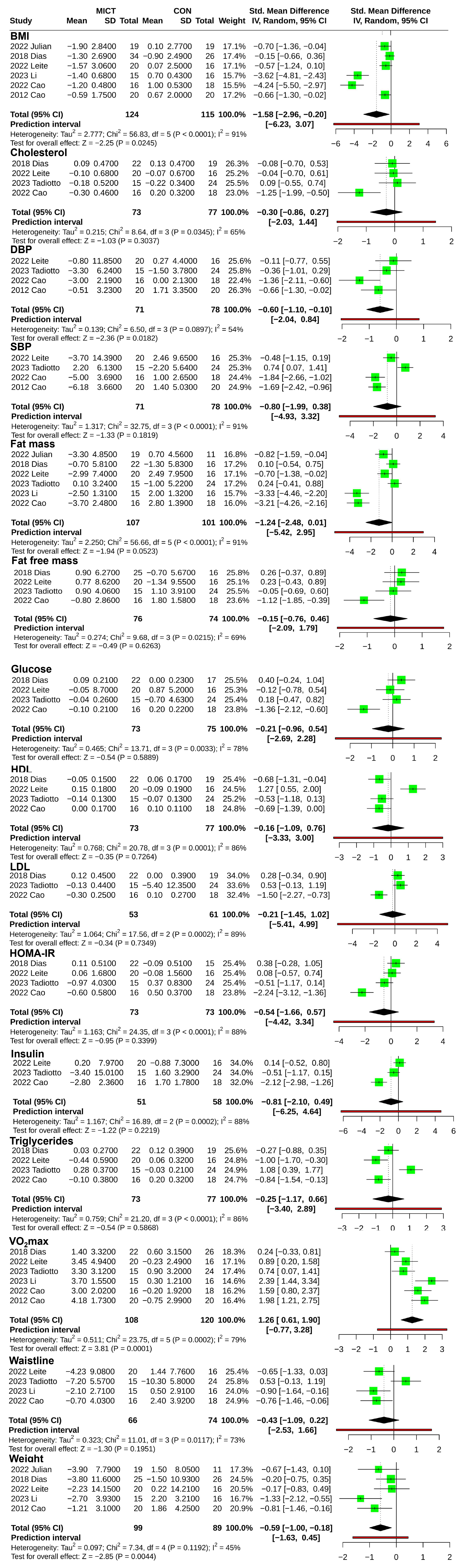

Supplement: Supplementary file 3 [file Image3.tif]

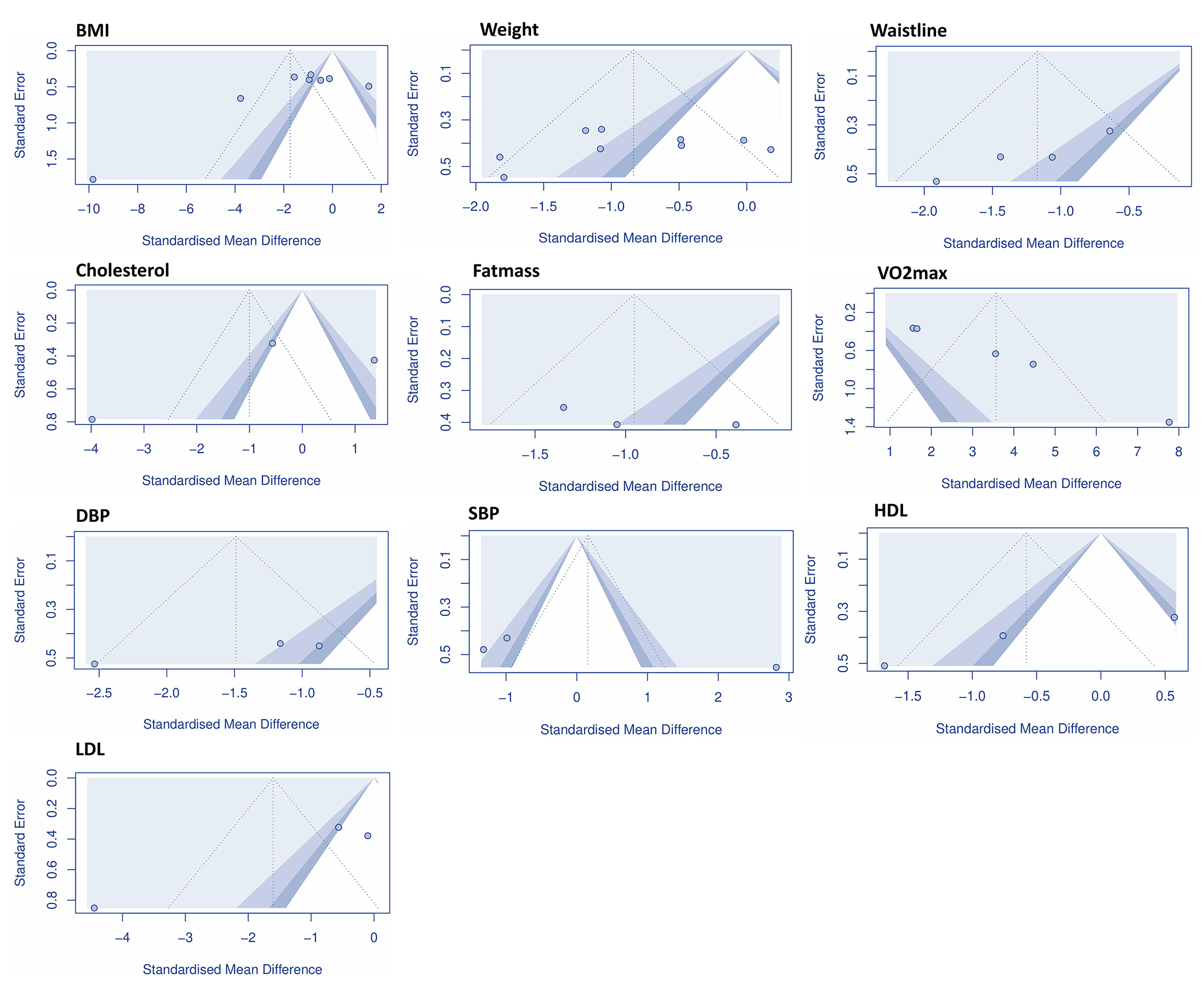

Supplement: Supplementary file 4 [file Image4.tif]

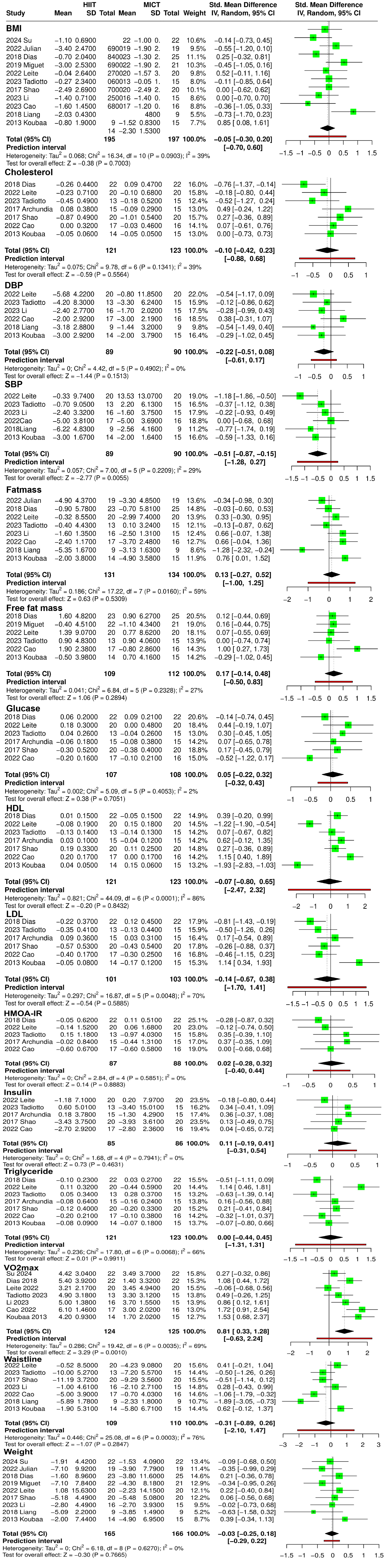

Supplement: Supplementary file 6 [file Image2.tif]

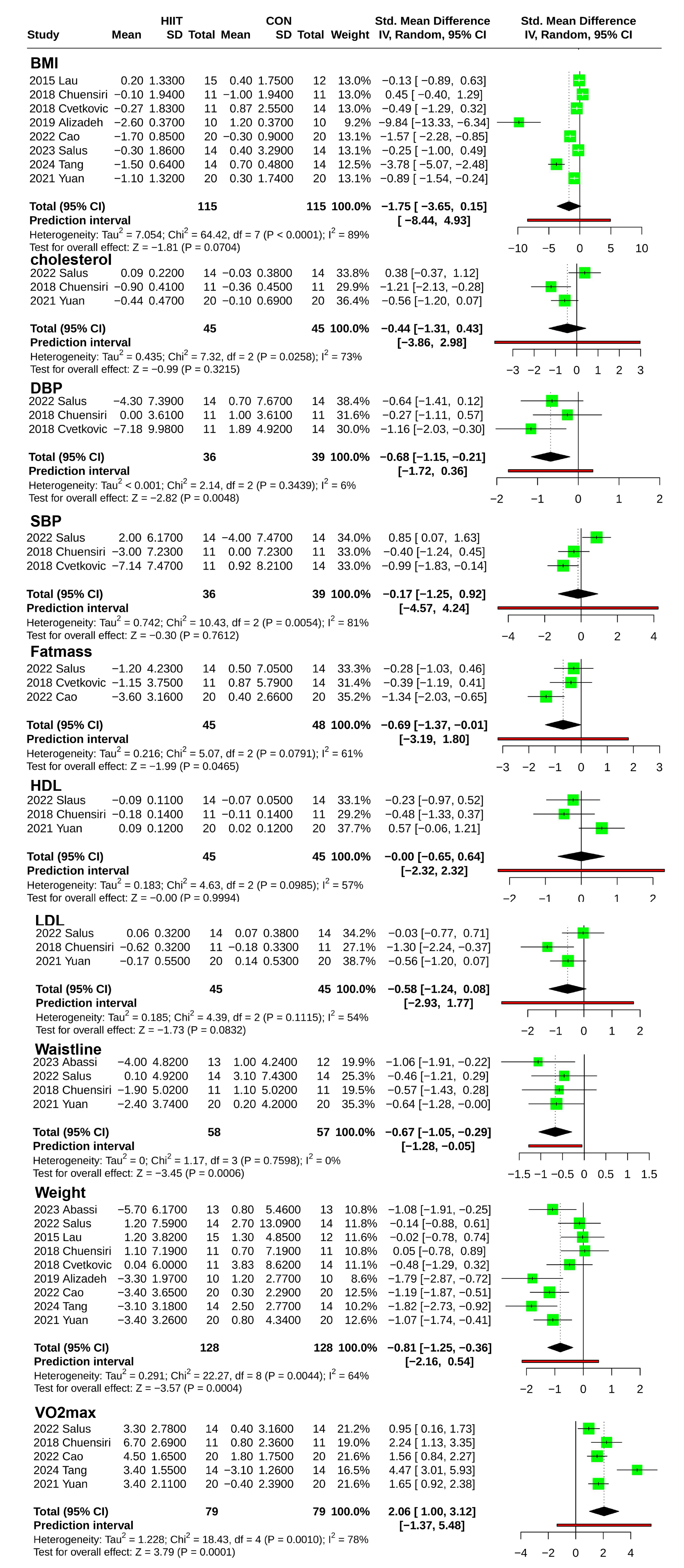

Supplement: Supplementary file 7 [file Image1.tif]

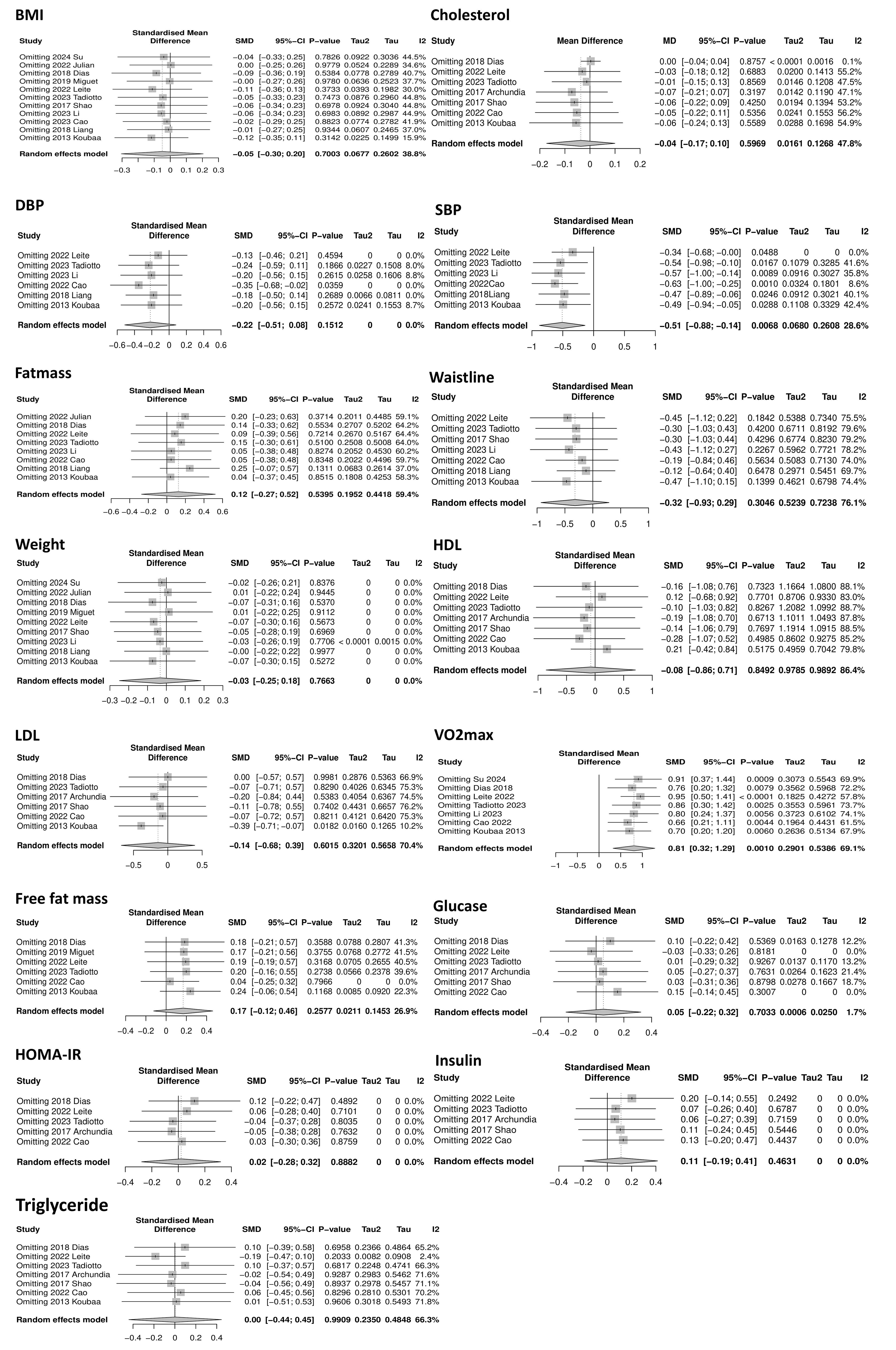

Supplement: Supplementary file 8 [file Image10.tif]

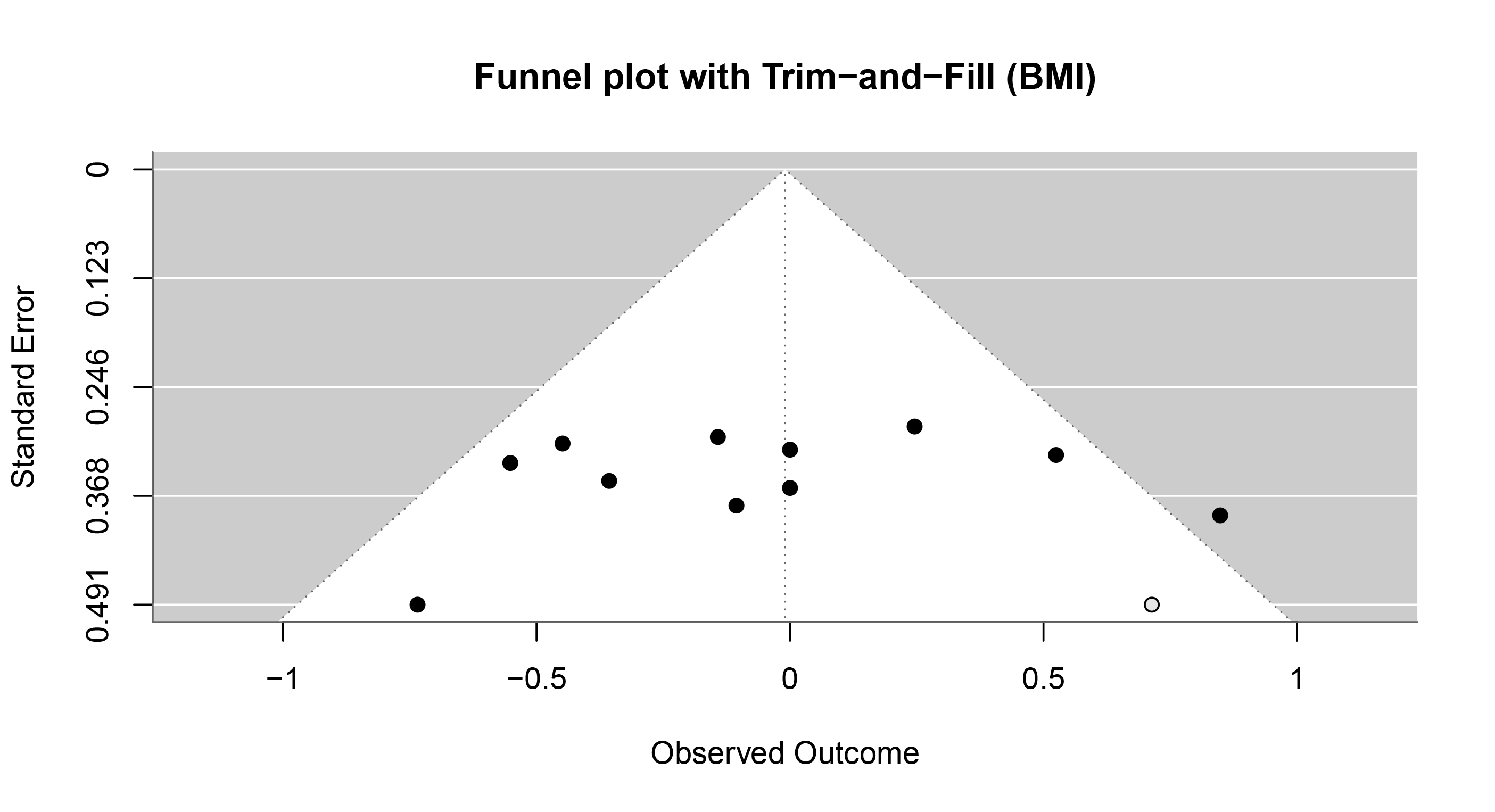

Supplement: Supplementary file 9 [file Image7.tif]
